# Antigen presentation, autoantibody production, and therapeutic targets in autoimmune liver disease

**DOI:** 10.1038/s41423-020-00568-6

**Published:** 2020-10-27

**Authors:** Andrea Kristina Horst, Kingsley Gideon Kumashie, Katrin Neumann, Linda Diehl, Gisa Tiegs

**Affiliations:** 1 Institute of Experimental Immunology and Hepatology, University Medical Center Hamburg-Eppendorf, Martinistraße 52, D-20251 Hamburg, Germany; 2 Hamburg Center for Translational Immunology, University Medical Center Hamburg-Eppendorf, Martinistraße 52, D-20251 Hamburg, Germany

**Keywords:** liver tolerance, autoimmune liver disease, antigen presentating cell, Autoimmunity, Immunological disorders

## Abstract

The liver is an important immunological organ that controls systemic tolerance. The liver harbors professional and unconventional antigen-presenting cells that are crucial for tolerance induction and maintenance. Orchestrating the immune response in homeostasis depends on a healthy and well-toned immunological liver microenvironment, which is maintained by the crosstalk of liver-resident antigen-presenting cells and intrahepatic and liver-infiltrating leukocytes. In response to pathogens or autoantigens, tolerance is disrupted by unknown mechanisms. Intrahepatic parenchymal and nonparenchymal cells exhibit unique antigen-presenting properties. The presentation of microbial and endogenous lipid-, metabolite- and peptide-derived antigens from the gut via conventional and nonconventional mechanisms can educate intrahepatic immune cells and elicit effector responses or tolerance. Perturbation of this balance results in autoimmune liver diseases, such as autoimmune hepatitis, primary biliary cholangitis, and primary sclerosing cholangitis. Although the exact etiologies of these autoimmune liver diseases are unknown, it is thought that the disruption of tolerance towards self-antigens and microbial metabolites and lipids, as well as alterations in bile acid composition, may result in changes in effector cell activation and polarization and may reduce or impair protective anti-inflammatory regulatory T and B cell responses. Additionally, the canonical and noncanonical transmission of antigens and antigen:MHC complexes via trogocytosis or extracellular vesicles between different (non) immune cells in the liver may play a role in the induction of hepatic inflammation and tolerance. Here, we summarize emerging aspects of antigen presentation, autoantibody production, and the application of novel therapeutic approaches in the characterization and treatment of autoimmune liver diseases.

## Introduction

Autoimmune liver diseases (AILDs), such as autoimmune hepatitis (AIH), primary biliary cholangitis (PBC), and primary sclerosing cholangitis (PSC), are immune-mediated liver injuries that are characterized by lymphocyte infiltration into the liver, increases in circulating immunoglobulins, elevated liver enzymes, the generation of autoantibodies, and genetic risk factors, such as HLA loci associations that predispose individuals to developing AILDs. In addition to AILDs, graft-versus-host disease after liver transplantation is an example of dysregulated hepatic immune homeostasis.^1,2^

AIH, which is characterized by interface hepatitis, is frequently associated with other autoimmune disorders (e.g., celiac disease, rheumatoid arthritis, and ulcerative colitis). In PBC and PSC, immune-mediated injury occurs in the bile ducts. PSC is also frequently accompanied by inflammatory bowel diseases. In PBC, small interlobular bile ducts are affected and present as nonsuppurative, destructive cholangitis. In PSC, the medium-sized intra- and extrahepatic bile ducts are affected, causing characteristic multilayered onion-skin fibrosis and multifocal bile duct obliteration.^3,4^ All three AILDs share a progressive clinical course, which can ultimately lead to liver fibrosis, cirrhosis and hepatocellular carcinoma or cholangiocarcinoma. To date, the most commonly used treatments are azathioprine in combination with corticosteroids for AIH and ursodeoxycholic acid (UDCA) for PBC and PSC.^4^ However, unstable or refractory disease, as well as frequent recurrence, prompt the need for liver transplantation as the last treatment option. The unsatisfactory therapeutic outcome in AILDs calls for the development of novel therapeutics. Among the research findings that have rapidly evolved from the study of liver disease are cell-based therapies using GMP-manufactured clinical grade Tregs,^5^ the role of regulatory B cells and the antibody-mediated depletion of B cells.^6^ Furthermore, the elucidation of noncanonical pathways for antigen presentation to MR1-restricted mucosa-associated invariant T (MAIT) cells or CD1d-restricted invariant natural killer T (iNKT) cells that bind to gut-derived and endogenous metabolites and lipids offers novel perspectives to understand the pathomechanisms in AILDs.^7^ In this review, we will summarize these emerging aspects of antigen presentation by professional and nonprofessional antigen-presenting cells (APCs) in the liver and their impacts on the immunological liver environment. The elucidation of novel canonical/conventional and noncanonical/nonconventional mechanisms of antigen presentation by MAIT cells, iNKT cells, and liver sinusoidal endothelial cells (LSECs), as well as trogocytosis and the cross-dressing of APCs, unveils new strategies for the development and application of novel therapeutics in AILDs. In addition, understanding of the functional role of (regulatory) B cells and Tregs and their recent successful therapeutic targeting in AILDs opens new strategies for the treatment of an otherwise refractory liver disease.

## Antigen presentation by alternative mechanisms and the effect on immune regulation in the liver

### Antigen presentation in the liver

The liver is a large reservoir of APCs and leukocytes, and intrahepatic immunity is biased towards tolerance.^8,9^ The impressive tolerogenic effect of the liver becomes evident after liver transplantation,^10^ which can result in spontaneous graft tolerance as opposed to the transplantation of other solid organs, such as the kidney, lung or heart.^11^ Indeed, a considerable proportion of patients can be weaned off immunosuppressive therapy after liver transplantation, whereas the transplantation of other solid organs requires life-long therapies. Nevertheless, 10–40 and 5% of liver transplant patients experience acute and chronic rejection, respectively.^12^ APCs in the liver are composed of dendritic cells (DCs) and macrophages (Kupffer cells, KCs), as well as nonmyeloid cells such as LSECs, hepatic stellate cells (HSCs), cholangiocytes, and hepatocytes (HCs).^8^ In addition, cellular transfer of MHC–peptide complexes via contact-dependent direct molecular transfer or extracellular vesicles can virtually enable any cell to act as an APC, albeit with different outcomes, as discussed below.

The licensing of APCs by CD8^+^ and CD4^+^ T cells occurs via MHC-I and MHC-II restricted antigen recognition, respectively.^8^ Hepatic APCs, such as DCs, express major histocompatibility complex classes I and II (MHC-I, MHC-II), as do KCs and LSECs, albeit at lower levels than DCs. MHC-I^+^ HCs, LSECs and DCs can directly present antigens to CD8^+^ T cells, whereas KCs, myeloid DCs, and LSECs express MHC-II and can additionally activate CD4^+^ T cells.^13^ Hepatic CD4^+^ T cell priming, however, predominantly results in the induction of tolerogenic Foxp3^+^ and Foxp3^+^ IL-10^+^ CD4^+^ Tregs.^14–17^ Additionally, intrahepatic priming of naïve CD8^+^ T cells results in the deletion of premature and nonantigen-specific CD8^+^ T cells. The outcome of T cell priming, however, depends on the overall antigen load, as well as on the context in which the MHC complexes are expressed: HCs express only MHC-I during homeostasis, which is also found on cholangiocytes in virally infected livers or biliary atresia.^18,19^ However, upregulation and de novo MHC-II expression on cholangiocytes and HCs can occur in virally infected or inflamed livers and elicit Th1 or Th2 effector responses.^19–21^ In clinical hepatitis (AIH, PBC, alcohol-induced hepatitis), HCs aberrantly express MHC-II, which is induced in the inflamed microenvironment by the inflammatory cytokine interferon gamma (IFNγ), and it was suspected that aberrant MHC-II expression could play a role in the pathomechanism of autoimmune hepatitis.^20^ However, transgenic mice with selective overexpression of MHC-II on hepatocytes did not display any predisposition or increased prevalence for autoimmune hepatitis.^21^ Interestingly, in the early stages of PBC, aberrant expression of MHC-II subregion genes (HLA-DP, HLA-DR, and HLA-DQ) can be detected on biliary epithelial cells, whereas in advanced disease, the expression of these genes decreases.^22^ In this context, it is worth noting that the expression of certain HLA-DR and HLA-DQ loci is associated with an increased risk for AIH and PBC, whereas other loci appear to convey protection.^23^ This finding indicates that specific but yet incompletely understood features of MHC-II-mediated antigen presentation may be integral in the immunopathobiology of AILDs.

## Trogocytosis and cross-dressing

Trogocytosis is causally involved in the pathogenesis of AIH, PBC, drug-induced liver injury, and steatohepatitis, and it has been observed during hepatitis B (HBV) and C (HCV) virus infections. In general, trogocytosis describes the cell–cell contact and T cell receptor (TCR)-dependent membrane transfer of peptide-loaded pMHC-I and pMHC-II complexes between T cells and professional or unconventional APCs. Of note, the acquisition of pMHC-II complexes by effector T cells (Teffs) or Tregs impacts their activity. Both cell types can acquire pMHC-II complexes, and for CD4^+^ T effector cells, this acquisition is associated with highly activated CD4^+^ T effector cells, whereas for Tregs, MHC-II-peptide ligands enhance their suppressive capacity.^24^ For Teffs, trogocytosis occurs continuously during cell cycle progression and marks highly proliferative cells with higher IFNγ production than pMHC-II^-^CD4^+^ effector T cells. In contrast, in activated Tregs, the acquisition of pMHC-II results in enhanced suppression of effector cell proliferation compared to that of pMHC-II^-^ Tregs in an antigen-specific manner. This enhanced suppression results from intimate Treg-Teff contacts in the immunological synapse that convey immediate effects of Treg-derived immunosuppressive mediators such as transforming growth factor beta (TGFβ), interleukin (IL-) 10, or cyclic adenosine monophosphate (cAMP).^24,25^ In addition, CTLA-4 on Tregs blocks CD80/86 signaling on APCs and subsequently downregulates CD28 costimulatory effects, which is required for effector T cell expansion.^26^

In addition to trogocytosis, intercellular protein transfer occurs via extracellular vesicles (EVs) and exosomes.^27,28^ However, the exact mechanism of intercellular protein transfer needs to be examined carefully and may not occur exclusively in one way.

Trogocytosis and the receipt of membrane proteins via exosomes generate MHC-dressed cells with transient noncanonical presentation of self or foreign antigens, which alters T cell activation and function. Important mechanisms that elicit immune regulation occur through trogocytosis, cross-dressing and the intercellular transfer of extracellular vesicles or exosomes that impose tolerance on effector cells (“tolerosomes”^29^). Cross-dressing refers to the transfer of preformed peptide:MHC class molecule complexes from one cell to another without further processing of the antigenic peptide.

Trogocytosis, also known as the intercellular transfer of peptide-loaded human leukocyte antigen (HLA) molecules via extracellular vesicles, can produce a broad spectrum of effects, such as the initiation and amplification of immune responses, the induction of anergy, the induction of regulatory cells, the desensitization of immunostimulatory receptors, the exhaustion of T effector cells, or the transmission of viral infection to previously uninfected cells.^30^

Hence, in general, antigen presentation can occur in the following direct and indirect manners: a. direct presentation, in which a viral antigen is presented by endogenous MHC-I molecules, such as by virally infected DCs that present viral antigen to CD8^+^ T cells; b. cross-presentation, such as the uptake of dying cells and the subsequent presentation of these exogenous antigens via endogenous MHC-I molecules to CD8^+^ T cells; c. cross-dressing, which occurs by intercellular MHC-I-peptide transfer from an APC or tumor cell to a DC via trogocytosis or exosomes and subsequently activates CD8^+^ T cells (this process does not require intracellular antigen processing by the DC); and d. MHC-II dressing, which occurs by intercellular MHC-II transfer (via trogocytosis/exosomes) of exogenous antigen-MHC-II complexes from DCs to neighboring DCs, CD4^+^ T cells, type 2 innate lymphoid cells (ILC2s) or natural killer (NK) and lymph node stromal cells.^27^ This contact results in T cell activation, and DCs/ILC2s express costimulatory molecules. ILC2s express CD80, CD86, and MHC-II and acquire MHC-II via trogocytosis, which induces a Th2 response in parasitic helminth infection.^31^ ILC2s are able to secrete the Th2 effector cytokines IL-4, IL-5 and IL-13.^32^ Regarding MHC-II, trogocytosis from DCs to mouse NK cells results in the suppression of CD4^+^ T cell responses, since NK cells are devoid of the costimulatory molecules CD80/CD86 and consequently do not elicit CD4^+^ T cell activation like DCs.^33^ Hence, after the acquisition of MHC-II, MHC-II-dressed NK cells suppress DC-induced CD4^+^ T cell responses.^33^

**Fig. 1:**
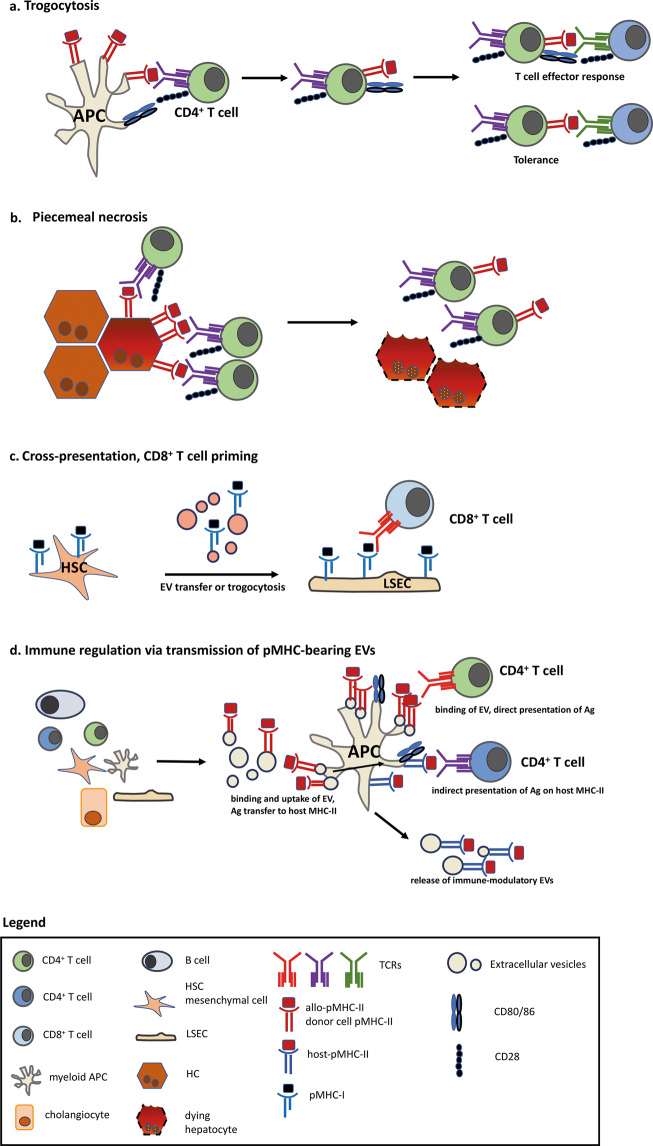
Examples of pMHC- and immunoregulatory molecule transfer via EVs or trogocytosis. **a** The transfer of peptide:MHC-loaded complexes by trogocytosis requires intimate cell-cell contact between APCs and T cells. During the transfer, only peptide:MHC complexes are transferred; additionally, costimulatory molecules, such as CD80/CD86, can be transferred from the APC to the T cell. Consequently, T cells can act as APCs, inducing the priming of naïve T cells. In the absence of costimulatory signals, tolerance results in T cell apoptosis and hyporesponsiveness. **b** Piecemeal necrosis describes the formation of an immunological synapse between peptide-loaded MHC-II^+^ HCs and CD4^+^ T cells with a cognate TCR that leads to peptide:MHC-II transfer onto CD4+ T cells. This process “eats away” part of the hepatocyte membrane and leaves behind dying HCs and CD4^+^ T cells with acquired peptide-MHC-II complexes. **c** HSCs transfer pMHC-I molecules to LSECs, which acquire cross-presentation abilities to elicit CD8^+^ T cell responses towards viral antigens. **d** Virtually all parenchymal and nonparenchymal cells produce extracellular vesicles/exosomes. Schematic representation of how pMHC-II complexes can be taken up and processed by APCs to generate alternate immune responses. First, the pMHC-II complex is taken up and presented by the APC, eliciting direct antigen presentation and CD4^+^ T cell priming in an immunological synapse that includes costimulatory molecules. Second, the MHC-II-bound peptide is taken up by the DC, processed and is consequently presented on host MHC-II molecules (blue) to elicit indirect antigen presentation and CD4^+^ T cell priming. Third, the pMHC-II complex is released by immunomodulatory EVs that carry the pMHC-II complex that confers APC properties to remote cells

In the liver, the majority of parenchymal and nonparenchymal cells are conventional or nonconventional APCs that are able to perform trogocytosis or produce extracellular vesicles/exosomes: HCs, cholangiocytes, LSECs, HSCs and liver-resident leukocytes.^34^ Trogocytosis in the liver was originally identified as “piecemeal” necrosis that was later renamed *troxis necrosis* and then was called trogocytosis (from ancient Greek for “to gnaw/to nibble”). Trogocytosis describes the formation of the immunological synapse between the T cell receptor (TCR) and antigen-presenting MHC-II-expressing hepatocytes, which leads to the acquisition of immune complexes by CD4^+^ T cells, and repeated, continuous “bites” into the hepatocyte induce hepatocyte necrosis^35–38^ (Fig. 1).

Later, the transfer of MHC molecules and MHC:peptide complexes has gained importance in liver immunology, such as the spreading and persistence of viral hepatitis, allograft acceptance and rejection, and it can lead to the aberrant formation of immunological synapses that foster autoimmunity. Furthermore, trogocytosis can be exploited by pathogens, such as *Entamoeba histolytica*, to acquire and display cell surface molecules from host cells to evade clearance by human serum components.^39^

A very recent study showed that MHC-I molecules can be transferred from HSCs to LSECs, and these LSECs cross-present viral antigens from infected HCs to retain and prime cytotoxic CD8^+^ T cells from the circulation^40,41^ (Fig. 1). Consequently, the local antiviral immune response is enhanced, and infected HCs are killed by activated cytotoxic CD8^+^ T cells via TNFα release. LSECs are able to express costimulatory molecules, such as CD80/86 and CD40, that can potentially support T cell activation.^42^ Bertolino et al. demonstrated that the liver induces antigen-specific activation of naïve CD8^+^ T cells, contrary to its tolerogenic role in inducing the deletion of premature and nonantigen-specific CD8^+^ T cells (graveyard hypothesis).^43^ The activation of naïve CD8^+^ T cells depends on the antigen load, and high antigen levels and the intensity of TCR stimulation induce activated memory CD8^+^ T cells. This process also partially depends on IL-2 secretion by naïve CD8^+^ T cells in the early phases of activation.^44^ In contrast, tolerance is induced if the antigen load is low.^45^ In addition, the priming of naïve T cells upregulates the expression of programmed death ligand (PD-L)1 on LSECs, which induces T cell apoptosis.^46^

As professional APCs, DCs have a prominent role in the induction of tolerance. Other than DCs from secondary lymphatic tissues, the majority of hepatic DCs constitute an immature subtype with comparably low expression of toll-like receptor 4 (TLR4), MHC-II and the costimulatory molecules CD80/CD86, which results in weak antigen presentation and poor priming of naïve T cells.^47^ Furthermore, intrahepatic DCs produce fewer inflammatory cytokines, such as IL-6 and IL-12p40, upon CpG stimulation than DCs in other tissues.^48^ The intrahepatic DC population consists of subsets that resemble immature, tolerogenic APCs that are resistant to maturation.^48–51^ To a large extent, intrahepatic DCs are plasmacytoid (B220^+^) DCs (pDCs) that produce tolerogenic cytokines, such as IL-10 and IL-27, in response to LPS stimulation.^48^ IL-27 signaling can then induce PD-L1 expression on hepatic pDCs via signal transducer and activator of transcription (Stat)3 signaling, leading to the generation of tolerogenic pDCs that are capable of inducing CD4^+^Foxp3^+^ Tregs in vitro.^48,52^ The importance of pDCs in tolerance has been highlighted recently in the context of MHC-mismatched mouse liver allografts^53^: intragraft DCs exhibited cross-dressing of MHC-I molecules from MHC-mismatched donors and induced host tolerance without the necessity for immune suppression. In humans, liver allografts, contrary to other solid organ transplants, are stable in one out of five patients without the need for immune suppression, and these recipients can eventually be weaned off immunosuppressive treatment.^54^ Crossed-dressed DCs (CD-DCs) play an important role in heart, kidney, islet and skin transplantation in that CD-DCs promote graft rejection.^55–57^ In contrast, in the liver, CD-DCs support graft tolerance, and the proliferation of alloreactive effector T cells is suppressed by CD-DCs.^53^ Liver allografts possess an unusually high level of PD-L1 that is expressed by allograft-derived nonparenchymal cells and CD-DCs, which supports the induction of Tregs but also suppresses donor-reactive host T cell proliferation and triggers apoptosis in alloreactive T cells.^53,58^ This tolerogenic effect is based on active signaling through triggering receptors expressed on myeloid cells-DNAX activating protein of 12 kDa (TREM-DAP12), which is required for the induction of IL-10 production.^53^ Ono *et al*. showed that graft-infiltrating CD-DCs exhibit activated DNAX-activating protein of 12 kD (DAP12) signaling, which is required to induce IL-10 production. Mice deficient in DAP12 exhibit a reduction in IL-10 production, which is crucial for the suppression of Th1 effector activation by APCs.^59^ Interestingly, DAP12^*-/-*^ mice exhibit acute allograft rejection, which is accompanied by a reduction in PD-L1/CD86 ratios on graft-infiltrating CD-DCs compared to those in WT liver grafts. This finding is noteworthy, since high ratios of PD-L1/CD86 on circulating pDCs are associated with increased circulating CD4^+^CD25^hi^ Tregs and transplant tolerance in human liver transplants.^60^ Hence, the authors suggest that PD-L1^+^ pDCs are cross-dressed and are implicated in mediating graft tolerance in their model.^53^ Furthermore, the presence of PD-L1^+^ CD-DCs correlates with the accumulation of PD-1^+^ T cell immunoglobulin and mucin domain-containing protein 3 (Tim3)^+^ effector memory T (Tem) cells in the allografts that stimulates cell death in Tem cells.^59^

## Soluble MHCs in tolerance and the diagnostic and therapeutic exploitation of cross-dressing and trogocytosis

In addition to membrane-bound peptide-loaded MHCs, soluble pMHCs play an important role in regulating tolerance induction; liver allografts are the source of significant amounts of soluble donor MHC class I molecules that remain detectable in the recipient´s circulation for extended periods of time.^61^ The fact that the liver is the largest solid organ leads to the hypothesis that the massive release of allogenic MHC-I molecules supports tolerance, since soluble MHC-I interacts with potentially alloreactive T cells in the absence of a costimulatory signal. These T cells are subject to apoptosis; hence, the release of soluble donor MHC-I constitutes an important tolerance mechanism.^62^

Therefore, the use of soluble pMHCs as well as their multimers to prevent allograft rejection or to modulate autoimmune reactions has gained considerable importance in recent years. For example, the generation of pMHC multimers for the detection and isolation of autoreactive T cells in autoimmune diseases has provided new tools to detect autoreactive T cells.^63^ The multimerization of pMHC molecules can overcome their inherent low-affinity binding to TCRs by increasing avidity and enable sufficient binding to the cognate self-^63^ and possibly alloreactive effector cells. Hence, the application of pMHC-multimer-based staining for low affinity TCR-expressing T cells enables the detection of autoreactive T cells that would normally not be detected via classic pMHC-tetramer-based staining.^63^

In addition, pMHC-II-coated nanoparticles have shown promising effects in preclinical applications for the amelioration of common autoimmune diseases in mice; nanoparticles coated with pMHC-II presenting disease-specific peptides from monospecific type 1 diabetes, EAE or collagen-induced arthritis can ameliorate disease activity and normalize glucose levels, neuromotor activities and joint inflammation.^64^ Furthermore, pMHC complexes on nanoparticles presenting autoantigens can reprogram autoantigen-specific effector T cells into CD4^+^CD25^-^Foxp3^-^ regulatory T cells (Tr1 cells) with disease-suppressing properties in experimental AILDs and the ability to induce regulatory B cells.^65^ The induction of these regulatory Tr1 cells is IL-10- and TGFβ-dependent, and their suppressive phenotype is transmittable via the transfer of tetramer^+^CD4^+^ T cells and portal/celiac lymph node-derived B cells from pMHC-II-treated donors. In addition, these transferred cells conveyed suppressive activity to proinflammatory hepatic and myeloid DCs and KCs. Interestingly, these Tr1 cells impaired autoimmunity in an antigen-dependent but not antigen-specific manner.^65^

**Fig. 2:**
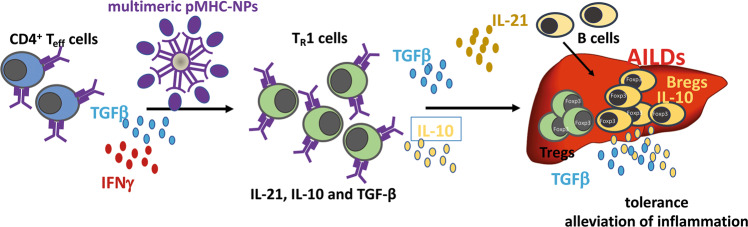
The application of multimeric pMHC-coated nanoparticles to induce tolerogenic TR1 cells, Bregs and Tregs. The application of multimeric pMHC complexes in the presence of IFNγ and TGFβ induces type 1 regulatory T cells (T_R_1 cells) that secrete IL-10 and TGFβ, as well as IL-21. This in turn aids the recruitment of B cells to the liver and induces tolerogenic Bregs and Tregs, which are both capable of secreting IL-10 and TGFβ, to impose tolerance and alleviate inflammation in AILDs

Based on known liver-enriched epitopes that are frequently detected in AILDs (i.e., anti-mitochondrial antibodies (AMAs), such as mitochondrial pyruvate dehydrogenase complex-E2 component (PDC-E2), or nuclear antigens (ANAs), such as the nuclear body-associated protein sp100 or the nuclear pore membrane protein gp120) and intracytoplasmic and Golgi-derived proteins such as formimidoyltransferase cyclodeaminase (FTCD), cytochrome P450 (CYPD2D6) or tropomyosin isoform 5 (hTM5) (please refer to^66^ for a detailed overview), pMHC-II could be applied in various mouse models to curtail disease activity with high organ specificity. In an impressive study, Umeshappa et al.^65,67^ compared organ versus disease specificity of disease-specific engineered pMHC nanoparticles that displayed PBC-relevant peptides (PDC-E2_166–181_/IAg7-NPs) and AIH-relevant peptides (CYPD_398–412_/IAg7-NPs), as well as PBC-relevant PDC-E2_166–181_/IAg7-NPs and AIH-relevant mFTCD_58–72_/IAg7-NPs and CYPD_398–412_/IAg7-NPs. The researchers found that engineered pMHC-conjugated nanoparticles loaded with all of these peptides expanded cognate CD4^+^ Tr1 cells and regulatory B cells and ameliorated necroinflammation and fibrosis, as well as ALT levels, in mouse models of AIH (Ad-hFTCD-infected NOD mice^68^) and PBC (NOD.*c3c4* mice^69^) (Fig. 2). These disease-reversing effects occurred without impairments in the desired immunity against metastatic allogenic tumors and pathogen insult, such as *Listeriae spp*., or vaccinia and influenza viruses.^65^

These findings demonstrate that molecular mimicry and epitope spreading may initially conceal the original antigens that cause AILDs. Although these mechanisms are involved in the pathology of autoimmune diseases, they may also offer novel perspectives for the development of AILDs therapeutics. Molecular mimicry emerges if foreign antigens, such as microbial or chemical substances, cause autoimmunity as a consequence of epitope similarity between foreign and self-peptides. As a result, autoreactive B and T cells are activated by antigens unrelated to the disease.^70^ Similarly, epitope spreading contributes to autoimmunity if the immune response and antibody repertoire diversify during the course of an infection. In this context, novel autoreactive B and T cell clones may emerge during an immune response that was originally mounted against a different antigen.^71^ Both mechanisms could therefore be exploited to test the potential of structurally related but disease-unrelated antigens to create diagnostic and therapeutic tools for AILDs.

## Extracellular vesicles

In addition to these direct effects on self and alloantigen presentation, extracellular vesicles can affect liver pathology in multiple ways. EVs are a heterogeneous group of lipid bilayer vesicles with different sizes, contents, and compositions. EVs range from 30–120-nm exosomes that emerge from the endosomal compartment and are released from multivesicular bodies into the extracellular space to 200–1000-nm microvesicles that are released from the plasma membrane of live cells and EVs released from apoptotic cells.^28^ Upon binding to a target cell, EVs can be taken up via endocytosis or remain tethered to their target cell surface and trigger intracellular signaling. In addition to the high surface levels of HLA-related molecules that enable efficient antigen presentation, EVs can also carry membrane-bound Fas ligand (FasL/CD95L) that conveys cell death signals upon binding to its receptor, Fas/CD95. In addition, EVs carry integrins, the asialoglycoprotein receptor and selectins, as well as soluble factors such as TGF-β and tumor necrosis factor alpha (TNFα).^72–74^ In this way, EVs are equipped with multiple tools to instruct lymphocytes and regulate immune responses (see Fig. 1).

Following clinical liver transplantation, the majority of circulating cells presenting donor MHC are cross-dressed.^75^ This indicates that T cell alloreactivity is triggered by recipient APCs that present intact donor MHC molecules on their surfaces, not necessarily by donor-derived passenger leukocytes (DCs) that present transplant-derived MHC molecules to naïve recipient T cells.^76^ This cross-dressing was first detected in the peripheral blood of patients after liver transplantation and has since been shown to stem from the transfer of molecules after direct cell-cell contacts or via EVs.^77^ Interestingly, a massive increase in the release of extracellular vesicles in the early phases after liver surgery has been observed, which may have a significant impact on cross-dressing and tolerance after liver transplantation, vide supra.^75,78,79^

EV release is strongly elevated after tissue insult and during tumor growth, and the lipid-, protein- and nucleic acid- (miRNAs, DNA, and RNA) content of their cargo is cell-specific. In that respect, EVs are useful biomarkers for a broad spectrum of inflammatory liver diseases, such as alcoholic hepatitis, hepatocellular carcinomas, liver fibrosis, and HCV. EVs in the serum of HCV patients contain a high level of miR-122, which is also found in mice, and show disease-specific accumulation of miR-134, miR-424 and miR-629-5p.^34^ MiR-122 was one of the first examples of a liver-specific miR that regulates the levels of plasma cholesterol and maintains systemic iron homeostasis.^80–82^

As known protein-based biomarkers associated with liver injury, EV cargo can contain high amounts of the (soluble) tetraspanin CD81, which is detected in the urine of rats with acute D-galactosamine hepatitis and in the serum of patients with HCV.^83,84^ Interestingly, CD81 is directly implicated in cellular HBV and HCV uptake since its interaction with claudin-1 and HCV glycoproteins catalyzes viral entry by enhancing membrane fusion.^85,86^ In addition, CD81 has additional functional roles regulating immune responses; as a costimulatory molecule, CD81 associates with CD4 and CD8 on effector T cells and can activate CD8^+^ T cells or prime CD4^+^ T cells.^87^ This hypothesis has interesting potential therapeutic implications for the treatment of HCV infections, since the costimulation of effector T cells via CD28 and CD81 acts additively^87^ in T cell activation and could support host defense.

Furthermore, EVs transmit HBV and HCV infection from infected to uninfected HCs, and EVs participate in viral replication.^88,89^ In addition, EVs from patients with HBV and HCV can exert both immunosuppressive and antiviral effects; in HBV, for example, EVs can impair NK cell activity and survival and downmodulate CD107a expression and IL-12 secretion, and these EVs show prominent surface expression of PD-L1, which induces T cell exhaustion.^88^ In this way, EVs from HBV patients can corrupt antiviral host defense.^88^ On the other hand, EVs can promote antiviral responses, as antiviral IFNα mediates the transfer of antiviral proteins through the release and internalization of EVs by HCs, which is initiated by nonparenchymal cells in the liver, such as LSECs and macrophages.^90^

In fatty liver diseases, such as models of high-fat diet feeding, human nonalcoholic fatty liver disease (NAFLD) and nonalcoholic steatohepatitis (NASH), EV release increases with disease severity and the increase in ALT levels, which activates inflammatory macrophages.^91,92^ Indeed, lipotoxicity stimulates EV release by HCs.^92^ In liver fibrosis, the levels of connective tissue growth factor (CTGF, CCN2), a key factor in the initiation of fibrosis, are elevated. In healthy livers, the endogenous reduction in CCN2 expression is mediated by miR-214, which is subject to control by Twist Family basic helix-loop-helix transcription factor 1 (Twist1), which is a key regulator of HSC activation.^93^ In fibrotic livers, the levels of miR-214 are decreased, thereby enhancing CCN2 expression. The expression of CCN2 can be controlled by exosomes that carry the transcription factor Twist1. These exosomes are released by HSCs and HCs and are subsequently engulfed by HSCs to exert transcriptional control of CCN2.^94^ In fibrotic livers, a reduction in Twist1^+^ exosomes enables the profibrotic activation of HSCs via an increase in CCN2 levels.^94^

In summary, naturally derived and modified EVs or engineered peptide-coated dextran- or pegylated iron oxide nanoparticles are novel and powerful tools to manipulate antigen presentation or educate liver-resident APCs, including HCs and cholangiocytes, and effector T cells. In the future, fine-tuning these technologies could aid in cell-specific hepatic delivery of therapeutic cargo. Notably, intravenous (i.v.) administration and biodistribution tracking revealed that the liver is the principal recipient site of i.v.-administered EVs.^95,96^ Regarding the potential therapeutic applications of modified EVs, efficient hepatic delivery of miR-155 with successful targeting of HCs and macrophages has been reported.^97^ MiR-155 expression is associated with inflammation and fibrosis induction in alcoholic steatohepatitis and after MCD feeding, and *miR-155*^*-/-*^ mice exhibit attenuated hepatic steatohepatitis and fibrosis induction.^97,98^

In addition, EVs are attractive biocompatible tools to deliver therapeutics to the liver, since they can be “transfected” to carry therapeutic miRNAs and RNAi or can be alternatively loaded with chemotherapeutics, given that they are able to present specific addressins on their surface, such as CXCR3 or CCR6, that could also be used to enhance liver targeting.^34^

## Autoantibodies, Bregs and their role in autoimmune liver disease

### Autoantibodies in autoimmune liver diseases

In autoimmune diseases, autoantibodies often develop before any clinical symptoms can be detected.

Autoantibodies in AILDs are rarely disease- or organ-specific and can also emerge in several nonautoimmune liver diseases, such as chronic viral hepatitis, NASH and drug-induced liver injury. Additionally, the aberrant presentation of intracellular or nuclear antigens on the cell surface is suspected to be a trigger for autoantibody production. This kind of ectopic presentation of autoantigens is associated with the pathogenesis of several autoimmune diseases.^99^

While autoantigens with diagnostic value for AIH and PBC could be discovered (also refer to the diagnostic scoring system by the EASL), these autoantigens are less relevant for the diagnosis of PSC, since cholangiography is the principal diagnostic method.^6,100,101^ It has been a persistent challenge to identify disease-specific antigens for autoimmune liver diseases that are of clinical and diagnostic relevance and that provide information on disease etiology. The autoantigens the autoantibodies bind to are often not organ-specific and exhibit significant cross-reactivity to unrelated antigens. In addition, highly variable antibody titers are observed in the course of the disease.^6,66,101^ Hence, the prognostic value of autoantibodies in AILDs is unclear, with some exceptions, such as α−LKM1 antibodies in AIH (target antigen: CYP2D6), α−ANA antibodies in PBC (target antigen: gp210), and α−GP2 IgA in PSC (target antigen: GP2). Additionally, α−ANA antibodies, which bind to the lamin B receptor, are of prognostic value in PBC. These autoantibodies point towards an increased incidence of HCC. Moreover, anti-centromere antibodies are indicative of a poor prognosis.^102,103^

Three AIH subtypes have been identified according to the emergence of different autoantibodies. AIH type 1 is the most predominant form of AIH in adults and children, and α−ANA and anti-smooth muscle actin (α−SMA) antibodies are prevalent. Of these, α−SMA antibodies are associated with the degree of hepatic inflammation.^104^ In AIH type 2, anti-liver-kidney microsomal type 1 (α−LKM1) antibodies have been detected, as well as α−LMK type 3 antibodies and/or anti-liver cytosol type 1 (α−LC1) antibodies. A more aggressive form of AIH, classified AIH type 3, is connected with the emergence of anti-soluble liver antigen/liver pancreas (α−SLA/LP) antibodies.^101,105–107^ In AIH type 3, α−SLA/LP is an IgG antibody that exhibits the highest specificity among all AIH-related antibodies and binds to the O-phosphoseryl-tRNA:selenocysteine tRNA synthase (SepSecS), which exhibits overlapping epitopes with CD4^+^ T cell epitopes. T-cell-mediated recognition of overlapping epitopes has also been reported for α−ASGPR- and α−CYP2D6 antibodies and is associated with T cell-mediated injury of hepatic parenchymal cells.^108–111^

In PBC, the hallmark anti-mitochondrial antigen (AMA) IgM antibody has been detected.^112^ Until now, it has been unclear whether this antibody is pathologically relevant. The α−AMA antibody binds to various epitopes in the pyruvate dehydrogenase complex, as well as the 2-oxo-acid dehydrogenase complex and branched-chain 2-oxo acid dehydrogenase complex pyruvate dehydrogenase E2 (PDC-E2). Contrary to IgM and anti-AMA antibodies, however, the prevalence of IgG3 and IgGA antibodies targeting AMA is indicative of severe disease.^113^ IgA is the major Ig isoform in the gut and is involved in mucosal protection, and secretory IgA is involved in regulating the gut microbiota composition, the transfer of antigens to DCs located in gut-associated lymphatic tissues, and dampening inflammation in response to pathogens.^114,115^ In addition, anti-sp100, anti-Kelch-like 12 and anti-hexokinase 1 antibodies are present in PBC, but the correlation of these antibodies with disease or prognosis is unknown.^101^

Furthermore, overlapping syndromes, molecular mimicry and epitope spreading hamper the identification of the original antigen that initiates the immune response, since pathogen infections and therefore microbial antigens are initiating epitopes and may drive the development of autoimmune disease. Hence, it is not surprising that inflammatory bowel diseases (IBDs) are common comorbidities in hepatic autoimmune diseases, especially in PSC.^116–118^ Approximately 70% of patients with PSC exhibit underlying IBD, especially ulcerative colitis, whereas 5% of patients with ulcerative colitis develop PSC. Intestinal inflammatory conditions foster *leaky gut syndrome* that enhances the concentration of microbial antigens in the portal circulation and can trigger hepatic inflammation.^119^ PSC is mostly associated with mild pancolitis, and in addition to the presence of p-ANCA, α−ANA or α−SMA antibodies, antibodies against biliary epithelial cells (BECs) have been detected, which are associated with adverse outcomes.^120–122^ Anti-BEC antibodies are of particular interest in PSCs, since Ig fractions from patients with anti-BEC antibodies induce TLR4/9 expression in BECs. The presence of LPS or CpG stimulates the secretion of inflammatory cytokines, such as IL-1β, IL-6, IL-8, IFNγ, TNFα, GM-CSF and TGFβ, that support effector cell polarization, as well as the CCR6 ligand CCL20 .^123,124^ Furthermore, activated BECs were shown to produce Th17-polarizing cytokines, and inflammatory infiltrates in diseased livers contain CCR6^+^CD4^+^ and aryl hydrocarbon receptor (AhR)^+^CD4^+^ naïve T cells with the potential to differentiate into Th17 effector cells.^124^ Furthermore, the binding of IgA antibodies to glycoprotein 2 (α−GP2 antibodies) is associated with PSC and indicate large bile duct involvement, increased risk for cholangiocarcinoma, and mortality. Elevated α-GP2 IgA levels in PSC patients correlates with secretory IgA concentrations, which are indicative of increased bacterial translocation and immune dysregulation.^101,125^ Moreover, α-GP2 IgA was initially described in the context of severe Crohn´s disease.^126^ The prevalence of IgA class antibodies in autoimmune liver disease strengthens the important connection between intestinal and hepatic immunity. Furthermore, in an attempt to characterize novel and unique antigens in PBC and PSC, the cells secreting disease-relevant antibodies were screened by antibody profiling of liver samples of PBC and PSC patients. Infiltrating B cells were identified as CD19^+^CD27^+^CD38^hi^CD138^-^ plasma blasts and differed from plasma cells (CD19^+^CD27^+^CD38^hi^CD138^+^). Liver-infiltrating IgM-positive plasma cells exhibited lower abundance in PSC than in PBC. In the antibody-producing cells derived from liver samples from PBC and PSC patients, liver-infiltrating cells were shown to produce antibodies with unique reactivities, revealing antigen specificity for nucleolar protein 3 and hematopoietic cell-specific Lyn substrate 1 in PSCs and PDC-E2 and hexokinase 1 in PBC.^127^

### Autoreactive B lymphocytes

In addition to their canonical immunoregulatory roles, such as antibody production, antigen presentation and the induction of T cells, autoreactive B cells can trigger the secretion of proinflammatory cytokines and T cell activation, leading to the differentiation of pathogenic effector T cells.^6^ Moreover, autoreactive B cells can inhibit Treg and regulatory B cells (Bregs or B10 cells in mice).^6^ Furthermore, antibody-secreting plasma cells are abundant in inflammatory infiltrates in patients with AIH.^128^ Approximately 10% of the inflammatory infiltrates around portal tracts are B cells, which were shown to be responsible for autoantibody production, the cross presentation of antigens, and the production of inflammatory cytokines.^6^ Through IL-21 secretion, B cells contribute to autoimmune disease. IL-21 is associated with autocrine and paracrine activation and increases in plasma cells and follicular helper T cells (Tfh cells) and correlates with disease severity.^129–131^ IL-21 levels in the sera of patients with AIH and the corresponding mouse model (neonatally thymectomized mice with systemic knockout of PD-1) are elevated. IL-21 is also secreted by activated splenic Tfh cells and drives CD8^+^ T cell proliferation and activation.^131^ Activated Tfh cells, in turn, can actively contribute to the development of spontaneous AIH in mice; after neonatal thymectomy in PD1^-/-^ mice, aberrant Tfh cell generation is observed, which induces AIH. In this model, activated splenic effector cells migrate to the liver in a CCR6-CCL20-dependent fashion and induce hepatocyte injury.^131^ The CCR6-CCL20 axis is known to recruit and position CXCR3^+^ Th17 effector cells from human and murine peripheral blood around portal ducts in diseased human or murine livers with inflammation, both in acute and in chronic liver injury models (concanavalin A-mediated liver injury and carbon tetrachloride-mediated liver injury).^132^ These data show that autoimmune liver disease is not only induced by local, intrahepatic immune cell activation but can also be stimulated by splenic/peripheral T cells.

### Regulatory B cells (Bregs)

**Fig. 3:**
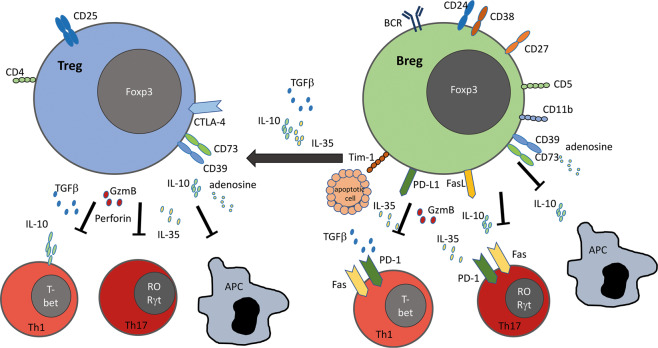
Tregs and Bregs exhibit overlapping immunoregulatory functions. Tregs and Bregs share functional similarities and present an overlapping spectrum of suppressive markers, such as Foxp3, CD39, CD73 IL-10, IL-35, and TGFβ, as well as GzmB, which exhibits cytotoxicity towards Th1/Th17 effector cells. Tregs can also secrete cytolytically active perforin. Altogether, immunosuppressive cytokines (IL-10, IL-35, TGFβ), as well as the secretion of cytolytic molecules (GzmB, perforin), suppress Th1 and Th17 effector cell polarization and activity, as well as antigen presentation by APCs. Interestingly, Bregs can induce Tregs via the secretion of TGFβ, IL-10 and IL-35. Tregs express the high-affinity IL-2 receptor CD25 and the signature marker CTLA-4. Bregs express the characteristic surface markers CD24, CD38, CD27, and CD5. CD11b contributes to immune suppression by downmodulating T cell receptor activation. In addition, Bregs induce Th1 and Th17 effector cell death via the expression of FasL, as well as by inducing cellular exhaustion through the expression of PD-L1, which binds to PD-1 on target cells. The Breg marker Tim-1 enhances Breg IL-10 expression after binding to apoptotic cells

Regulatory B cells were first discovered in the context of delayed type hypersensitivity^133^ and have only recently become a research focus in autoimmune liver diseases. Compared to Tregs, Bregs or Bregs with the capacity to produce IL-10 (B10 cells) exert similar immunosuppressive effects (Fig. 3). Although Bregs or CD19^+^CD1d^hi^CD5^+^ B10 cells^134^ are capable of producing immunosuppressive IL-10, TGFβ and IL-35,^135,136^ only a fraction of CD19^+^CD1d^hi^CD5^+^ B10 cells produce IL-10. In the murine spleen, for instance, IL-10-producing Bregs constitute approximately 1–2% of total splenocytes, and in the human liver, the total B cell population contributes only approximately 6% to the total liver lymphocyte population.^137^ However, diverse murine models of autoimmune disease, such as encephalomyelitis (EAE),^138^ chronic colitis,^139,140^ collagen-induced arthritis,^141^ type 1 diabetes,^142^ systemic lupus erythematosus,^143^ and allograft rejection/transplantation tolerance,^135,144^ were exacerbated after B cell depletion or impaired Breg functions. In general, autoimmune diseases are characterized by inappropriate activity or expansion of Th1 and Th17 effector cells, with a concomitant reduction in Treg frequencies or alterations in their suppressive potential.^145–147^ Bregs are capable of suppressing such effector T cells and inducing Tregs in both humans and mice. In addition to IL-10 and TGFβ, Bregs are capable of secreting granzyme B and thereby killing T effector cells. Bregs can also express FasL and PD-L1, which can induce cell death or exhaustion in CD4^+^ T effector cells and induce Tregs.^148,149^ Two murine B10 populations that produce IL-10 have been identified: a CD19^+^CD24^hi^CD38^hi^ and a CD24^hi^CD27^+^ Breg population.^150^ Functionally, CD19^+^CD24^hi^CD27^+^ Bregs were more suppressive than the CD24^hi^CD38^+^ subtype, as they expressed higher levels of IL-10, TGFβ, and GzmB.^150^ Interestingly, the CD19^+^CD24^hi^CD27^+^ Bregs also express integrin α4β7, which indicates their potential for gut homing and a different anatomical site of action. Additionally, IL-10^+^ Bregs express PD-L1 and the ecto-ATPase CD39, likely rendering these cells capably of suppressing other immune cells via PD-1 or the generation of immunosuppressive adenosine.^151,152^

Quantitative and functional alterations in CD24^hi^CD38^hi^ B cells have been observed in patients with PBC. CD24^hi^CD38^hi^ B cells were elevated compared to healthy controls, which correlated with the level of cholestasis,^153^ suggesting that these CD24^hi^CD38^hi^ B cells may not have regulatory functions. Indeed, when analyzed for cytokine production and surface marker expression, these B cells produced high levels of IL-6 and IL-12 and exhibited reduced IL-10 and Tim-1 expression.^153^ Interestingly, in PBC patients, Tim-1 levels were markedly downregulated in immature/transitional CD19^+^CD24^hi^CD38^hi^ B cells that are normally capable of expressing high levels of IL-10.^153,154^ In addition, the frequencies of peripheral Th1 and Th17 cells were elevated and correlated with increased CD19^+^CD24^hi^CD38^hi^ B cell frequencies.

The majority of IL-10^+^ Bregs express Tim-1,^138,155^ and Tim-1 has been identified as an essential marker for the promotion and maintenance of IL-10 production in Bregs.^138^ Tim1-deficient B cells lose the capability to produce IL-10 and thus upregulate the inflammatory cytokines IL-6, IL-12 and IL-1β. As a consequence, Tim1^-/-^ B cells promote Th1/Th17 cell differentiation but inhibit the generation of Foxp3^+^ Tregs or Tr1 cells. Thus, Tim-1 could be exploited to induce IL-10^+^ Bregs. Indeed, ligation of Tim-1 by antibodies and subsequently triggering Tim-1 signaling elicits tolerogenic B cells.^156,157^ Hence, Tim-1^-/-^ Bregs promote EAE, whereas Tim-1^+^ Bregs inhibit EAE.^138,155^ This finding confirms the protective role of Tim-1-expressing Bregs in autoimmune disease. In addition, Tim-1 manipulation can be used to elicit tolerance or rejection; a high-affinity Tim-1 antibody (monoclonal antibody (mAb) 3B3), enhanced Th1 and Th17 cell induction, accompanied by functional Treg deprogramming in an allograft rejection model and a model of allergic asthma.^158,159^ Treg deprogramming refers to the functional impairment of Tregs that is induced after the application of mAb 3B3 and results in reductions in critical Treg markers, such as Foxp3, glucocorticoid-induced TNF receptor family–related protein (GITR), CTLA-4, and IL-10. In addition, the capacity of these αTim1 antibody-treated Tregs to suppress the proliferation of CD4^+^ effector cells was markedly reduced.^158^

In contrast, a low-affinity anti-Tim1 antibody (mAb RMT1-10) was used to modulate allograft tolerance of pancreatic islet grafts, and Tim1^+^ B cells could transfer tolerance since tolerogenic cytokine expression (IL-4, IL-10) and the induction of Bregs were enhanced.^156^ This low-affinity antibody (mAb RMT1-10) has previously been shown to be effective in inhibiting EAE and supports cardiac allograft survival.^160,161^

In addition to Tim-1, CD11b expression may correlate with B cell suppressive functions. After mice were immunized with a mixture of liver antigens, Bregs regulated effector CD4^+^ T cell activation and proliferation, which depended on CD11b expression, which is induced by B cell-derived IL-10.^162^. Consequently, adoptive transfer of Bregs or the depletion of (effector) B cells ameliorates experimental inflammatory and autoimmune diseases, such as Sjögren´s syndrome, Myasthenia gravis, collagen-induced arthritis or preclinical transplantation models.^163–167^ In addition, as described above, targeting Tim-1 on Bregs with antibodies offers promising immunotherapeutic pathways. Tim-1 binds to phosphatidylserine residues on apoptotic cells, which was exploited by Gray *et al*. in a preclinical mouse model of arthritis. The authors described that i.v. administration of apoptotic cells induced IL-10 secretion in splenic B cells and therefore fostered Breg induction.^167^

### Targeting B cells in autoimmune liver injury

The pathogenic role of B cells in autoimmune diseases has been established in diabetes type 1, SLE, and arthritis.^6^ By presenting self-antigens, producing autoantibodies and secreting IL-21, B cells can contribute to the induction and maintenance of autoimmune diseases. Consequently, targeting B cells may provide novel strategies in the treatment of autoimmune diseases and in the liver.

### B cell depletion

The goal of B cell targeting in autoimmune liver diseases is to eliminate B cells, which present autoantigens and subsequently prime CD4^+^ effector T cells to induce HC injury via the production of inflammatory effector cytokines. Rituximab, a chimeric mouse-human mAb with binding specificity for CD20, was the first anti-CD20 mAb for the depletion of B cells and is used for the treatment of hematological malignancies (especially lymphomas), as well as a broad spectrum of systemic autoimmune disorders.^168–170^ In autoimmune liver diseases, rituximab (anti-CD20 antibodies^171,172^) was first administered to pediatric and adult AIH patients who were refractory or intolerant to prednisone and azathioprine treatment, and this has proven to be successful in some cases. Moreover, in adult AIH, rituximab treatment resulted in increased tissue and/or circulating Treg numbers.^172^ Additionally, a recent report showed that rituximab could improve liver enzymes and reduce the dose of prednisone in 22 difficult-to-treat AIH patients without severe side effects,^173^ which was associated with biochemical, histological and immunological remission and a significant reduction in flares in more than 70% of the patients in a two-year follow-up.^173^

In PBC patients, alterations in the B cell repertoire have been observed. Analysis of B cell clonal diversity showed a marked reduction in clonal diversity and somatic hypermutations in class-switched sequences, which were more prominent in PBC patients with cirrhosis.^174^ Additionally, significant increases in IgM-producing cells, together with elevated IgM serum levels, were observed in PBC patients. Standard treatment of PBC patients with ursodeoxycholic acid (UDCA) increased clonal diversity and reduced Ig levels but had no effects on the observed reduction in somatic hypermutation.^174^ Thus, under UDCA treatment, PBC patients exhibit insufficient responses with regard to these B cell parameters. In contrast, treatment of PBC patients with rituximab decreased serum levels of total IgG, IgM, and IgA, as well as anti-mitochondrial autoantibodies. Additionally, 1 year after rituximab treatment, patient-derived B cells showed reduced IgM secretion after stimulation.^175^ Furthermore, CD20^+^CD27^+^ memory B cells decreased transiently in rituximab-treated PBC patients, which was accompanied by an increase in CD4^+^CD25^+^ T cells, elevated *FOXP3* and *TGFβ* mRNA expression levels, and decreased *TNFα* mRNA levels in CD4^+^ T cells.^175^ Thus, in PBC patients, targeting B cells may provide clinical benefits.

### Anti-BAFF/BAFFR treatment

In addition to targeting B cells in experimental models or treatments, B cell growth factors are the focus of therapeutic targeting. B cell activating factor of the TNF family (BAFF) is secreted by innate myeloid-derived immune cells, such as neutrophils, macrophages, monocytes and DCs, as well as by T cells and B cells. BAFF acts as a trimeric ligand to its cognate receptors (B cell *a*ctivating factor of the TNF-*f*amily *r*eceptor (BAFFR), B cell maturation Ag (BCMA), and transmembrane activator and calcium modulator and cyclophilin ligand interactor (TACI) and induces the development, maturation and survival of B cells.^176,177^ BAFF expression is stimulated by the type 1 interferons IFNγ, IL-10 and G-CSF, as well as by TLR4 and TLR9 activation.^178,179^ Excess levels of BAFF are thought to contribute to the development of autoimmune diseases. BAFF can support T cell-independent activation of B cells that express BAFF receptors, such as BAFFR and TACI.^177^ Both BAFF- and BAFFR-deficient mice exhibit impaired B cell maturation, decreased levels of IgG, poor T cell-dependent and -independent immune responses, and moderate allograft tolerance. In contrast, BAFF transgenic mice develop T cell-independent lupus-like disease or rheumatoid-like arthritis.^180,181^ Interestingly, BAFF does not impair the deletion of high-affinity self-reactive B cells but instead enhances the propagation of low-affinity self-reactive B cells and protects self-reactive B cells from peripheral deletion.^182^ Furthermore, lupus-prone MRL/lpr mice exhibit reductions in IL-10^+^ Bregs but increases in BAFF expression and show preferential expansion of IL-10^-^ B cells in response to inflammation.^183^ The selection of autoreactive B cells over tolerogenic IL-10^+^ Bregs results from BAFF-dependent proinflammatory NFκB- and JNK-mediated signaling.^183^ Furthermore, the application of a TACI-IgG fusion protein in SLE and a mouse model of experimental allergic encephalitis induced the expansion of IL-10^+^ Bregs and ameliorated disease.^183^

Although there is a lack of studies of anti-BAFFR antibodies in mouse models of autoimmune liver disease, ongoing clinical trials use anti-BAFFR antibodies (Ianalumab, VAY736). The first randomized placebo-controlled trial in adults with AIH with incomplete therapeutic response or intolerance to standard treatments (AMBER study: ADCC Mediated B cell dEpletion and BAFF-R Blockade; NCT03217422, phase 2/3) targets BAFFR together with the deletion of B cells. Further trials that use B cell-directed therapies include studies with Belimumab (Benlysta®), a neutralizing anti-BAFF antibody, for third-line treatment of AIH; so far 2 patients with refractory AIH were treated, and complete responses and remission were achieved.^184^

These applications and results for repurposed B cell-targeted therapies demonstrate that B cells do have a significant and previously underappreciated impact on programming the immunological microenvironment in the liver. The identification and characterization of autoantibody-secreting B cells will help to identify additional disease-relevant antigens. As B cells can exert both beneficial and detrimental effects in AILDs, it challenging to balance B cell-targeted therapies to enhance the expansion and function of Bregs and eliminate autoreactive effector B cells at the same time.

## Cholangiocytes as nonconventional antigen-presenting cells

Cholangiocytes produce and secrete bile acids and serve as a mucosal barrier to bile fluid. As such, they protect the hepatic parenchyma from the deleterious effects of potentially toxic bile acids and microbial products and the invasion of microbes, which can gain access from the gut.^185^ To date, there have been no reports that cholangiocytes can present peptide antigens in classic MHC molecules. However, cholangiocytes are able to present lipid antigens via the MHC-like molecule CD1d and present bacterial metabolites derived from the B2 (riboflavin) pathway via the MHC-related receptor MR1. Riboflavin metabolites and riboflavin derivatives activate MAIT cells, whereas the products of folic acid degradation compete with riboflavin derivatives and block MAIT cell activation.^186^ Lipids bound to CD1d are recognized by restricted (i)NKT cells and lead to (i)NKT cell activation.

### Cholangiocytes are nonconventional APCs that activate MR1-restricted MAIT cells

For a more detailed and translational understanding of the functional role of antigen presentation by cholangiocytes, it is important to elucidate mechanisms by which cholangiocytes present antigens and activate immune cells. In particular, PSC has a significant connection to IBD. Hence, microbial challenges and the defense mechanisms of the epithelial barrier in both the intestine and the liver are crucial for understanding the connection between gut leakage and biliary and hepatic inflammation.

**Fig. 4:**
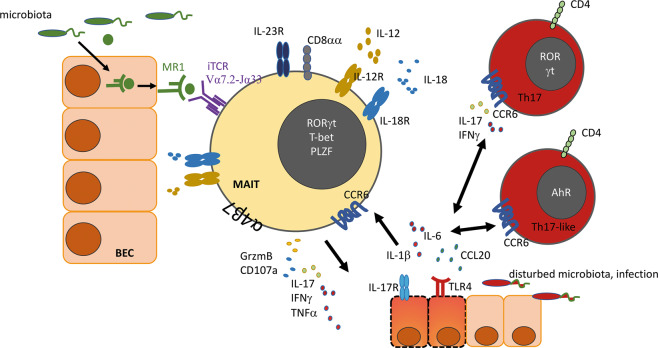
Bidirectional crosstalk between BECs and MAIT cells. MAIT cells express an invariant TCR (Vα7.2-Jα33), as well as the receptors for IL-12, IL18 and IL-23 and the signature transcription factors RORγt, T-bet and PLZF. The major MAIT cell population expresses CD8αα. Furthermore, liver targeting of MAIT cells is controlled by the expression of CCR6 and integrins, such as the gut and liver homing integrin α4β7. The MR-1-restricted presentation of microbial vitamin B metabolites by BECs to the invariant TCR induces the activation of MAIT cells, which triggers the release of effector molecules, such as GzmB, CD107a, IL-17, IFNγ, and TNFα. Consequently, AhR^+^ Th17-like and RORγt Th17 effector cells are induced. These cells, in turn, express CCR6 and release IL-17 and IFNγ, which induce BEC inflammation. In conjunction with infection due to pathogenic shifts in the microbiota, BECs release IL-1β, IL-6 and CCL-20 in response to LPS-induced TLR-4 activation. This, in turn, enhances biliary injury and perpetuates ductal inflammation. In addition to MR-1-restricted TCR activation, MAIT cells can be activated by high local concentrations of the inflammatory cytokines IL-12 and IL-18, which are secreted by activated macrophages

MAIT cells are innate CD3^+^CD161^+^ T cells with a conserved invariant T cell receptor alpha chain (Vα7.2-Jα33/Jα20/Jα12 in humans, Vα19-Jα33 in mice).^187,188^ MAIT cells resemble effector memory cells, and they recognize vitamin B metabolites from the pathogenic and commensal microflora on the highly conserved MHC-I-related protein 1 (MR1). MAIT cells produce gut epithelia-protective IL-17 and IL-22. In general, MAIT cells are activated by TLR-stimulated APCs or infected APCs that harbor different bacteria, viruses or yeast.^189^ MAIT cell activation occurs in a TCR-dependent fashion or by inflammatory cytokines (such as IL-7, IL-12, IL-15 and IL-18, as well as type I interferons), and both mechanisms can act in synergy. TCR-dependent stimulation of MAIT cells rapidly induces perforin, FasL, granzyme B/H, RORγt, PZLF, TNFα, or CD40L, as well as IL-17A expression (Fig. 4). In contrast, MAIT cell activation induced by inflammatory cytokines triggers a slow response, with the induction of T-bet and IFNγ.^190–194^ Interestingly, MAIT cells can be activated by type I interferons, which suggests that during human viral infections, such as HCV infection, these cells may participate in regulating the hepatic immune microenvironment.^192,194^ MAIT cells were identified by a modified human MR1 tetramer, which revealed that these cells are predominantly CD8^+^ cells (CD8a^+^CD1b^-/low^).^188^

In humans, MAIT cells constitute up to 20–40% of intrahepatic T cells.^190,195,196^ Interestingly, the frequency of liver-resident MAIT cells (LI-MAIT cells) is higher in healthy livers than in chronically diseased livers (NASH ALD, PSC, and PBC), indicating that these cells may have a protective role in preventing liver disease. Moreover, in liver diseases, such as ALD, NASH, PBC and PSC, not only are MAIT cells reduced but CD8^+^ MAIT cells, which are normally the most abundant, are also decreased.^197^

In the liver, MAIT cells are located in the liver sinusoids in proximity to ducts of portal biliary tracts, which are in immediate contact with gut-derived bacterial antigens.^197^ Correspondingly, LI-MAIT cells express CCR6, CXCR6 and α4β7 integrin, which promote their recruitment and localization to the bile ducts in both normal and inflamed livers.^132,198^ Additionally, intrahepatic MAIT cells express CXCR3, the receptor for the IFNγ-induced ligands CXCL9 and CXCL10, and LFA-1 and VLA-4 integrins, which supports the hepatic recruitment of these cells from peripheral blood. Furthermore, MAIT cells express high levels of IL-18R, which allows for intrahepatic activation in inflammatory conditions, such as by KCs in the hepatic microenvironment. Importantly, LI-MAIT cells also express MR1, and contact between LI-MAIT cells and *E. coli* triggers MR1-dependent degranulation and IFNγ secretion in LI-MAIT cells.^197^ If MAIT cells from the peripheral blood and LI-MAIT cells are cocultured with BECs that had been stimulated with bacteria, MAIT cells become activated in an MR1-dependent but cytokine-independent manner, thereby inducing the secretion of granzyme B (GzmB) and IFNγ and the upregulation of CD40L.^197^ In PSC patients, a decline in MAIT cells was associated with impaired MAIT cell response to *E. coli*, which resulted in a reduction in GzmB, CD107a, TNFα, IFNγ and CD69 expression in MAIT cells. This finding is of note, since it stresses the importance of MAIT cell dysfunction and the potential of these cells to protect the integrity of the biliary epithelial barrier.^199^ Furthermore, in patients with alcoholic liver disease and alcohol-related cirrhosis and underlying gut leakage syndrome, MAIT cells in the peripheral blood declined, and there were reductions in IL-17 and GzmB and CD107a levels, as well as a reduction in the expression of the MAIT-related transcription factors RORγt and PLZF.^200^ These findings support the role of MAIT cells in epithelial barrier protection, which is often breached and results in severe bacterial infections in patients with PBC and PSC.

In patients with NAFLD or NASH, PD-1 was also shown to be upregulated on LI-MAIT cells. Furthermore, LI-MAIT cells produce IL-17A, which is known to activate HSCs and enhance the progression of liver fibrosis.^201–203^ Böttcher *et al*. showed that in AILDs, long-term stimulation of MAIT cells in the presence of IL-12 and IL-18, as well as bacterial antigens, fostered MAIT cell exhaustion due to chronic antigen exposure, although these cells expressed activation markers (CD38, HLA-DR and CD69). The exhausted phenotype was evident by the upregulation of PD-1, TIM-3, and CD39, with simultaneous increases in IFNγ expression. This finding indicates that these MAIT cells are phenotypically exhausted but exhibit continuous production of inflammatory IFNγ.^203^ Moreover, a reduction in MAIT cell survival was observed; however, although MAIT cells decreased in number over time, they exhibited increased secretion of the profibrogenic cytokine IL-17A. Importantly, IL-17A derived from MAIT cells of inflamed livers with autoimmune disease and NASH was able to induce the proliferation of primary human HSCs, accompanied by the induction of the expression of profibrogenic signature genes, such as SMA, LOX1, TIMP-1 and Col1, as well as the inflammatory cytokines IL-1β, IL-6, IL-8, and CCL2.^203^ Contrary to these findings, predominantly activated MAIT cells were found in explants of cirrhotic livers by Hedge et al., and MAIT cells from cirrhotic patients with and without alcoholic liver disease exhibited increased expression of CD25 and CD69, as well as increased secretion of GzmB and IL-17.^204^ MAIT cells were located in the mesenchymal space within the fibrotic septa and exhibited profibrogenic activity. Consistently, *Mr1*^*-/-*^ mice were resistant to fibrosis, whereas mice with endogenously elevated levels of MAIT cells (transgenic Vα19TCRTg mice) exhibited exacerbated fibrosis in a carbon tetrachloride model of liver fibrosis or bile duct ligation. However, there were no significant differences in ALT levels or CD4^+^ and CD8^+^ T lymphocytes, B lymphocytes, neutrophils, macrophages, and dendritic cells between these murine lines. This finding enhances the importance of alterations in MAIT cell activity in the development of biliary cirrhosis.

**Fig. 5:**
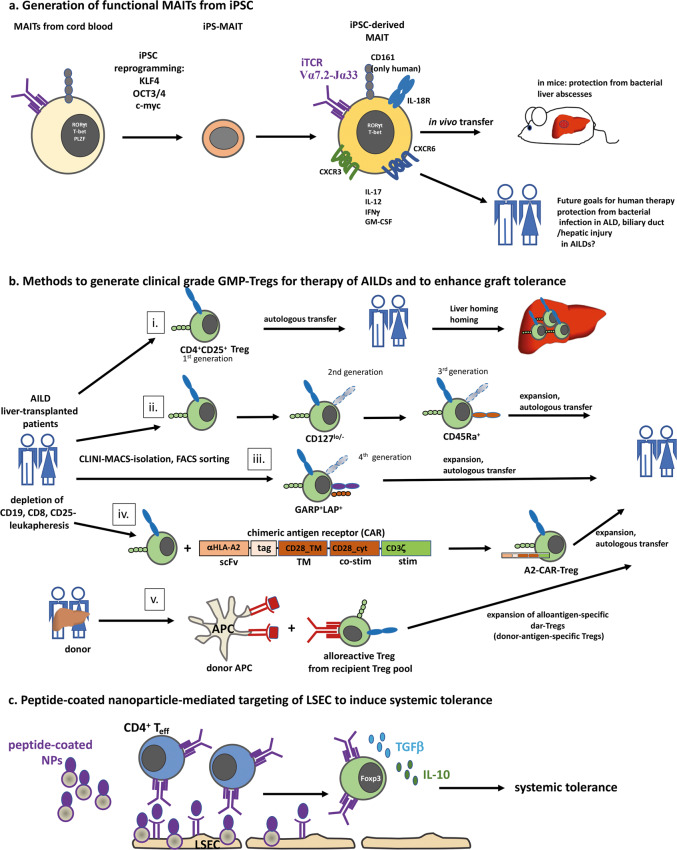
Preclinical and clinical cell-mediated therapies for AILDs. **a** Generation of functional MAIT cells. Functional MAIT cells can be generated from cord blood after iPSC induction by the established iPSC transcription factors Krüppel-like 4 (KLF-4), Oct3/4, and c-myc. The resulting iPS-derived MAIT cells express typical MAIT markers, such as iTCR (Va7.2-Ja33), CD161, and IL-18R, and characteristic cytokines, such as IL-17, IL-12, IFNγ, and GM-CSF. In addition, the MAIT cell transcription factors RORγt and T-bet are expressed by iPSC-derived MAIT cells. Upon in vivo transfer, the liver-targeting receptors CXCR3 and CXCR6 are expressed. In vivo, functional MAIT cells protect mice from bacterial liver abscesses. In humans, functional MAIT cells alleviate hepatic inflammation by protecting against dysbiosis in alcoholic liver disease and can potentially be applied in AILDs, such as PBC or PSC. **b** Methods to generate clinical-grade GMP-Tregs. Clinical grade GMP-Tregs can be obtained after leukapheresis from AILD patients and liver transplant recipients. Following leukapheresis and the depletion of CD8^+^, CD19^+^ and CD25^-^ lymphocytes with magnetic bead-assisted enrichment via a CLINI-MACS^TM^-system, Tregs are sorted by flow cytometry: (i) 1^st^ generation clinical-grade CD4^+^CD25^+^ Tregs, (ii) 2^nd^ generation CD4^+^CD25^+^CD127^lo/neg^ Tregs, and 3^rd^ generation CD4^+^CD25^+^CD127^lo/neg^CD45Ra^+^ Tregs, or (iii) 4^th^ generation CD4^+^CD25^+^CD127^lo/neg^GARP^+^LAP^+^ Tregs. Additionally, Tregs can be equipped with a chimeric antigen receptor to convey antigen specificity and efficient cell stimulation and induction (iv). Here, an example of the generation of A2 CAR Tregs that convey tolerance in GvHD is shown. (v) Donor-antigen-specific Tregs can be generated by coculturing donor-derived APCs with polyclonal Tregs from the recipient to yield donor-antigen-specific Tregs (dar-Tregs). Tregs are enriched before transfer into the patient (i.), or Tregs are expanded ex vivo using IL-2, retinoic acid, rapamycin and anti-CD3 and anti-CD28 antibodies, summarized here as “expansion” (ii–v), before the cells are transferred back into the patient. **c** Peptide-coated nanoparticle-mediated targeting of LSECs to induce systemic tolerance. Nanoparticles coated with autoantigenic peptides are taken up by LSECs and displayed on pMHC-II complexes to potentially autoreactive CD4^+^ Teff that express a cognate TCR. This interaction results in the induction of Tregs and systemic tolerance

Hence, MAIT cells exert both pathogenic and protective roles in liver disease, which depends on their particular tissue localization, cytokine profile, and the stage and chronicity of the disease. MAIT cell frequencies and alterations in phenotype correlate to disease onset and severity. MAIT cells are able to discriminate between commensal bacterial colonization and bacterial infections since chronic TCR-mediated activation occurs predominantly in the presence of inflammatory cytokines.^205^ In alcoholic liver disease, which is associated with severe gut leakage, MAIT cell dysfunction is connected to alcohol-induced alterations in the composition of the gut microbiota.^200^ Thus, chronic exposure of MAIT cells to bacterial antigens drives MAIT cell exhaustion, which results in the exacerbation of bacterial infections in patients with severe alcohol-induced hepatitis. These data show that therapeutic manipulation of the gut flora and the restoration of the gut epithelial barrier support the restoration of MAIT-induced epithelial protection. Since AILDs are also associated with IBDs, the restoration of MAIT cell function by modulating the gut microbiota or cell therapy with induced pluripotent stem cell (iPSC)-derived MAIT cells could be applied for the treatment of AILDs.^206^ Wakao *et al*. explored the redifferentiation of human MAIT-like cells from iPSCs. The researchers showed that iPSC-derived MAIT cells expressed the MAIT cell-specific markers TCR Va7.2 and CD161, as well as IL-18R and RORC^207^ (Fig. 5A). Upon restimulation with *E. coli*-induced monocytes, iPSC-MAIT cells produced effector cytokines (IL-12, IFNγ, GM-CSF, and IL-17), showing that these cells were functionally intact. Adoptive transfer of these engineered iPSC-MAIT cells was able to convey protection against liver abscesses from mycobacteria (*M. tuberculosis*).^207^ Importantly, adoptively transferred iPSC-MAIT cells expressed the gut/liver homing receptors CXCR3 and CXCR6, demonstrating that the expression of these liver addressins was inducible. Taken together, these findings impressively show that the adoptive transfer of iPSC-MAIT cells harbors the potential to restore MAIT cell function (Fig. 5A).

### Cholangiocytes activate CD1d-restricted iNKT cells in autoimmune liver disease

iNKT cells belong to CD4^+^ or CD4^-^ type I or type II subsets according to their T cell receptor composition and the self or nonself lipid ligands presented by the highly conserved MHC-like molecule CD1d.^208,209^ Type I iNKT cells express an invariant Vα14Jα18 chain in mice (Vα24Jα18 in humans) together with a less variable Vβ chain (humans: Vβ 11 chain^210^) and constitute up to 30% of intrahepatic lymphocytes in mice.^209^ When activated by their ligand alpha-galactosylceramide (αGalCer), Type I iNKT cells exert innate-like immune responses. Type II NKT cells express more diverse alpha and beta TCR chains. iNKT cells in murine livers largely resemble Th1-like cells and are capable of rapidly producing inflammatory Th1 and Th17 effector cytokines.^211^

Primary human cholangiocytes and both human and murine cholangiocyte cell lines express the MHC-class I-like molecule CD1d, which enables these cells to present endogenous and microbiome-derived lipid antigens and αGalCer to (i)NKT cells and to activate these cells in vitro.^212^ In liver disease, the expression of CD1d differs among disease etiologies. In PBC patients, CD1d is expressed on small bile duct cholangiocytes^213^ and is upregulated in early-stage PBC^213^ but can be lost in late-stage PBC,^212^ whereas in PSC patients, CD1d is expressed on the epithelia of the larger bile ducts. This finding indicates that iNKT cells may play a role in cholestatic and fibrotic liver diseases, although it is not clear whether iNKT cells protect against or exacerbate disease. For example, in the murine AIH model of concanavalin (Con) A-induced hepatitis, liver injury is mediated by TNFα, and iNKT-mediated cytotoxicity is enhanced by the autocrine secretion of IL-4, leading to the subsequent release of perforin and GzmB, as well as FasL-mediated HC death.^214–216^ In contrast, in αGalCer-mediated liver injury, IL-17 neutralization exacerbated liver damage, which was demonstrated by the influx of neutrophils and inflammatory monocytes,^215^ indicating that NKT-derived IL-17 mediates protective effects after αGalCer injection.^214,215^ In a mouse model of oxazolone-induced cholangitis, peribiliary infiltration of T cells, as well as Ly6G^-^ and Mac2^+^ myeloid cells, was dependent on CD1d expression and the activation of NKT cells.^217^ Using *Cd1d*^*-/-*^ mice or anti-CD1d blocking antibodies, biliary disease was markedly reduced or abolished, respectively.^217^ Further studies also showed that initial iNKT cell activation by microbial antigens is a critical step in the development of chronic, T cell-triggered autoimmunity in small bile ducts in murine models of PBC.^218^ Infection of mice with *N. aromaticivorans* bacteria induces chronic T cell-mediated autoimmunity and antibodies against microbial PDC-E2 in murine hosts, resembling human pathology in PBC. In this model, the induction of disease is dependent on molecular mimicry and CD1d-mediated activation of NKT cells that recognize bacterial cell wall components, highlighting the importance of the connection between microflora and biliary autoimmune diseases.

### Bile acid synthesis by cholangiocytes is modulated by inflammatory T cells

Direct antigen presentation by cholangiocytes to T cells in an MHC-restricted fashion can also lead to biliary injury.^219,220^ Transgenic overexpression of ovalbumin in cholangiocytes (ASBT-OVA) preferentially activates OVA-specific CD8^+^ T cells but not CD4^+^ T cells, leading to liver injury.^219^ Additionally, adoptive transfer of antigen-specific CD8^+^ T cells into *Mdr2*^*-/-*^*xK14-OVAp* mice, which express the OVA peptide in cholangiocytes, is sufficient to induce biliary disease.^220^ Antigen-specific CD8^+^ T cells localized in the peribiliary regions and were associated with the downregulation of enzymes involved in bile acid (BA) synthesis (cholesterol 7α-hydroxylase (Cyp7a1), sterol 12α-hydroxylase (Cyp8b1), sterol 27α -hydroxylase (Cyp27a1), and oxysterol-7α hydroxylase (Cyp7b1)) and an increase in BA secretion by HCs. Blocking IFNγ and TNFα with antibodies resulted in the upregulation of key enzymes involved in BA synthesis (Cyp7a1/Cyp8b1) and BA transporters,^220^ indicating that this change in BA synthesis depended on inflammatory cytokines and the presence of inflammatory CD8^+^ T cells. These data indicate that there is bidirectional communication between biliary APCs and inflammatory T cells that can influence BA metabolism. The authors hypothesize that IFNγ and TNFα can influence signaling of the master regulator of BA synthesis and homeostasis farnesoid X receptor (FXR),^221^ which enforces BA synthesis and hepatocellular BA secretion. Glaser *et al*. therefore suggested complimenting FXR-directed therapies for cholangitis with a T cell-targeted approach to restore the disturbed BA homeostasis.^220^

## Tregs in autoimmune liver diseases

Tregs are instrumental in maintaining immune tolerance by suppressing effector responses and supporting the resolution of immune effector responses during regeneration. Tregs actively suppress the proliferation and activation of (potentially) autoreactive CD4^+^ T cells and cytotoxic CD8^+^ T cells. This active suppression mechanism is the key to self-tolerance. In addition, clonal deletion of self-reactive lymphocytes is an important mechanism for the maintenance of tolerance; however, tolerance is not insurmountable, since autoimmunity can be stimulated by immunization in a healthy organism with self-antigens plus adjuvant.

In AILDs, Tregs show different changes in function or numbers in the peripheral blood and livers of patients, or these cells may even be functionally unaltered; in PSC, FOXP3^+^ Tregs are associated with polymorphisms in the IL-2Ralpha gene, which correlates with a reduction in Treg numbers in the liver and peripheral blood.^222^ Sebode et al. showed that effector cells from PSC patients exhibited increased proliferation capacity, regardless of whether they were suppressed by Tregs. Hence, if suppression capacities are normalized, Tregs from PSC patients exhibit lower suppression of Teff proliferation than those from healthy controls.^222^ In addition, Tregs from PSC patients produced less IL-10 and expressed lower levels of the TGFβ-tethering surface marker surface receptor glycoprotein-A repetitions predominant (GARP) than those from healthy controls.^222^ In contrast, in AIH patients, peripheral CD4^+^CD25^+^FOXP3^+^CD127^lo^ Tregs appear to be fully functional and not reduced in number.^223^ In contrast, intrahepatic frequencies of Tregs were increased proportionally in the inflammatory infiltrates.^223^ These data are controversial, however, since Ferri *et al*. and Buckner *et al*. showed decreased Treg numbers and compromised suppressor functions, depending on the activity of disease, or the resistance of effector T cells to suppression.^224,225^

In contrast, in patients with chronic HBV and HCV infection, as well as in patients with hepatocellular carcinomas, Treg numbers are increased in untreated patients, which compromises the expansion and effector function of antiviral CD8^+^ T cells.^226–229^ This effect could add to the chronicity of infection, since an efficient antiviral response is blocked.

In mice, Tregs are defined as CD4^+^CD25^+^Foxp3^+^ T cells, whereas in humans, this characterization is insufficient, since effector T cells can express Treg markers. Therefore, Tregs are defined as CD4^+^CD25^+^FOXP3^+^CD127^low^ T cells, since CD127 expression inversely correlates with FOXP3 levels.^230^ The CD4^+^CD25^+^Foxp3^+^CD127^low^ Treg population constitutes approximately 1–5% of liver-infiltrating lymphocytes in diseased livers.^231^

In liver disease, hepatic Tregs constitutively express CTLA-4, which is a target gene of Foxp3, and the ecto-ATPases CD39 and CD73, as well as the immunosuppressive cytokine IL-10.^231–233^ Furthermore, Tregs are induced in environments with low IL-2 concentrations, which can be produced by activated CD4^+^ and CD8^+^ T effector cells in inflamed tissues. Due to the expression of the high affinity IL-2Rα (CD25), these cells can rapidly sequester IL-2 from the environment and thereby impair the proliferation of effector T cells and NK cells.^234^ Furthermore, TGFβ suppresses the proliferation and effector functions of NK cells, such as IFNγ secretion, and prevents NK cells from enhancing missing self-recognition.^235^ Tregs downregulate the expression of costimulatory CD80/CD86 on DCs via CTLA-4 and increase the expression of indoleamine 2,3-dioxygenase (IDO).^236^

IL-2-mediated signaling initiates the master program that phosphorylates STAT5, which is essential for Treg proliferation, differentiation and survival, and induces the expression of the Treg master transcription factor Foxp3.^237^ Furthermore, functional TCR signaling is required for the maintenance of a Treg phenotype.^238^ In addition to IL-2, the induction of peripheral Tregs from naïve CD4^+^CD25^-^ T cells requires TGFβ to induce Foxp3 expression.^239^ Intrahepatic Treg numbers are increased in AILDs, paralleling the number of CD4^+^ T effector cells, and Tregs express high levels of CTLA-4, CD39 and IL-10. Tregs in inflamed livers express high levels of CD95 and are highly sensitive to FasL-mediated apoptosis, which occurs in IL-2-deprived inflammatory environments. Treg apoptosis can be induced by cholangiocytes, as well as HCs.^231^ In contrast, exogenously administered IL-2, however, can restore Treg survival.^240^ This finding indicates that biliary or hepatic inflammation can actively contribute to the decrease in intrahepatic Tregs in autoimmune diseases such as PSC or AIH. Intrahepatic Tregs are also capable of secreting GzmB and perforin, albeit to a significantly lower extent than intrahepatic CD8^+^ T cells.^231^ Furthermore, depending on their local cytokine microenvironment, Tregs exhibit plasticity and can convert into inflammatory Th1 or Th17 effector cells.^241,242^

As discussed above, the lack of disease-specific antigens for the majority of AILDs poses a challenge for the development of disease-specific agents or the isolation of antigen-specific Tregs. The exceptions are AIH type 2 and PBC, which exhibit known antigen specificity.^243,244^ Currently, the transfer of ex vivo-expanded Tregs (autologous donor-alloantigen-reactive Tregs, autologous polyclonally expanded Tregs) is under investigation for improving transplantation therapy or alleviating AILDs.^245–247^

Strategies to use Tregs in disease treatment include enriching Tregs and enhancing their function, such as the expansion and stabilization of Tregs by low-dose IL-2 therapy, or by the use of superagonists, such as anti-IL-2 antibody/IL-2 complexes, or muteins, which increase IL-2Ra receptor affinity. These superagonists have a longer half-life than native IL-2, as well as an increased affinity for IL-2Rα. In addition, supplementation with vitamins such as all-trans-retinoic acid and vitamin D have been used to promote Treg expansion.^5,248–254^ In addition, gene editing of Tregs has become an additional strategy for optimizing Treg-based therapies. Peripheral Tregs express CXCR3, which greatly enhances the hepatic recruitment of Tregs by intrahepatic synthesis of the cognate ligands CXCL9-11.^212^ In mice, the transfer of ex vivo-expanded CXCR3^+^ Tregs or intrahepatic Tregs expressing CXCR3 ameliorates the outcome in mouse models of AIH, such as ConA-induced immune-mediated hepatitis or in an experimental AIH type 2 correlate.^255,256^ As expected, the lack of CXCR3 in mice results in the exacerbation of ConA-induced hepatitis.^257^ Based on these findings, CXCR3-transfected Tregs were used experimentally in the liver, intestines, and lung to alleviate GvHD after bone marrow transfers.^258^ Here, the authors confirmed that GvHD was less pronounced in mice that had received CXCR3-engineered Tregs than in mice that received nontransfected Tregs and that these genetically engineered Tregs mainly targeted and localized in the liver.^258^ Furthermore, chimeric antigen receptor (CAR)-Tregs^259,260^ provide an attractive option to optimize Treg stability and prevent exhaustion by the introduction of costimulatory molecules, such as CD28 or CD137/4-1BB.^260,261^ The first CAR-Tregs contained an HLA-A2–specific CAR (A2-CAR) that was used to generate alloantigen-specific human Tregs.^259^ Leving´s group demonstrated that in a mouse model of GvHD, engineered CAR-Tregs provided more effective immunosuppression than transferred polyclonal Tregs. A2-CAR Tregs express an anti-HLA-A2 single chain Fv, followed by a tag, a CD8 stalk, a transmembrane and cytoplasmic coreceptor domain, and a C-terminal CD3ζ chain (Fig. 5b). A very recent report showed the optimization of an A2-CAR platform with Tregs that expressed a CAR including the intact CD28 transmembrane and cytoplasmic domain, and these cells were significantly more effective in suppressing GvHD than A2-CAR Tregs with cytoplasmic domains from 4-1BB, ICOS, CTLA-4 or GITR.^262^

In current therapies and translational approaches to developing disease-specific Tregs, it is desirable to target autoantigen- or alloantigen-reactive Tregs to the liver. In an attempt to develop disease-specific Tregs from AIH type 2 patients that displayed predisposing HLA-DR7 and/or HLA-DR3 alleles, Tregs were generated from cultures of peripheral blood mononuclear cell-derived T and B lymphocytes supplemented with IL-2, anti-CD3/CD28 antibodies, and AIH-2-specific CYP2D6 peptides.^243^ In subsequent cocultures with CYP2D6 peptide-pulsed semimature DCs, CD4^+^CD25^hi^ Tregs were selected for effector T cell suppression assays and the suppression of CD8^+^ cytotoxicity.^243^ Notably, these CYP2D6 peptide-specific Tregs expressed FOXP3 and the liver-homing receptor CXCR3 and showed efficient effector T cell suppression with concomitant reductions in the effector cytokines IFNγ and IL17. In addition, the suppression of cytotoxic activity by CD8^+^ T cells was observed.^243^ These findings show that antigen-specific Tregs can be generated for AIH type 2 and fulfill their suppressive roles.

Contrary to this antigen-specific approach, the expansion and therapeutic application of polyclonal Tregs is currently the only option for AIH type 1 or PSC patients.

### GMP production by Tregs for clinical applications in AIH

For GMP production and clinical applications, Tregs can be purified by leukapheresis from peripheral blood or umbilical vein blood, as well as from the thymus.^245,263^ Peripheral blood Tregs are enriched by a density gradient after leukapheresis. The 1^st^ generation of CD4^+^CD25^+^ Tregs was purified by magnetic microbead-aided depletion of CD8^+^ T cells and CD19^+^ B cells, with subsequent positive selection of CD25^+^ T cells (Fig. 5b). The second generation of Tregs was enriched by CD4^+^ selection and flow sorting for CD25^high^CD127^low/-^ cells, which yielded an increased proportion of FOXP3^+^ Tregs. Further selection of CD45Ra^+^ Tregs (third generation) selected for a proliferative population of Tregs. Expansion of the purified Tregs was performed with anti-CD3/CD28-coated beads with the addition of IL-2, rapamycin and retinoic acid. Autoantigen-specific Tregs may be selected by their LAP and GARP expression, as this population appears to be rich in autoantigen-specific Tregs, which can be expanded ex vivo (4^th^ generation Tregs).^245^ To date, Treg infusion has been used for the treatment of type 1 diabetes, GvHD and living donor liver transplantation.^264–267^ In addition, donor-alloantigen-reactive (dar)-Tregs can be generated by stimulating donor-derived APCs with polyclonal Tregs from the recipient to yield antigen-specific Tregs (Fig. 5b). This approach offers a promising strategy for the treatment of GvHD in (liver) transplant recipients.

For the development and evaluation of standardized GMP-grade Treg protocols for the treatment of AIH, Oo *et al*. recently showed in a proof of concept study (Autologous T-regulatory cell tracking after InfUsion in AutoiMmuNe Liver Disease (AUTUMN)-study) that autologous polyclonal GMP-grade nonexpanded CD4^+^CD25^high^ Tregs isolated by leukapheresis and closed automated sorting (CliniMACS^TM^) could induce stable remission in 4 AIH patients in remission, 3 of whom were cirrhotic. Importantly, 22–44% of the initially transferred Tregs were able to home to the liver and were detectable intrahepatically for up to 72 h after infusion.^246^ Tregs from the patients were functionally suppressive, expressed critical functional Treg markers such as CD39,^268^ CTLA-4^269^ and CXCR3 and were metabolically competent.^246^ Importantly, patients receiving this well-tolerated therapy with a single maximal dosage of 8.6 × 10^6^ Tregs did not show severe adverse events or stable disease over a 4-month follow-up period, although no significant changes in liver enzymes or Ig levels were detected.^246^

For Treg therapy, the timing and dose (acute, remission or relapse, chronicity of disease, fibrotic or nonfibrotic, metronomic vs. bolus therapy, numbers of Tregs to be transferred), as well as the maintenance of a stable and functional Treg phenotype in the inflammatory hepatic environment, need to be investigated carefully. The fact that autologous transfer of Tregs is well tolerated, even after the transfer of high numbers of cells, suggests that this cellular therapy is a promising option for future extended treatment of autoimmune diseases.

## LSEC-mediated T cell education

Blood, which permeates the liver, enters the hepatic circulation via the sinusoids. The liver sinusoids are lined with specialized cells called the LSECs, which constitute the vascular bed of the liver.^270^ LSECs are a phenotypically and functionally distinct subpopulation of endothelial cells, forming a thin repetitive but fenestrated layer that separates the space of Disse from the sinusoidal lumen.^8^ The liver is tailored to maintain a delicate balance between immunity against potential invading pathogens and tolerance of harmless antigens,^271^ and LSECs play important roles in these processes. The primary site of pathogen or antigen exposure is the sinusoids, in which KCs and LSECs are involved in sequestering antigens from incoming portal blood. To initiate an immune response, pattern-recognition receptors (PRRs) such as Toll-like receptors (TLRs) and scavenger receptors (SRs)^272,273^ on LSECs facilitate antigen uptake. Notably, LSECs express high levels of scavenger receptors such as the mannose receptor and stabilins, which have cell-autonomous roles in immune responses.^273,274^ Indeed, scavenger receptors have been shown to potentiate anti- and proinflammatory signaling and to interact with TLRs.^273^ Upon binding to extracellular ligands, scavenger receptors expedite the endocytosis of the cargo, which is routed into the endosomal pathway,^5^ suggesting that LSECs can regulate immunity via antigen presentation to T cells. LSECs and passenger leukocytes extensively interact in vivo in the sinusoids, promoting the recruitment of hepatic leukocytes.^275^ The recruitment of hepatic leukocytes by LSECs is largely driven by the constitutive expression of CD54 (ICAM-1), CD106 (VCAM-1), vascular adhesion protein (VAP)-1, CD44 and hyaluronan.^8^ Thus, the recruitment of passenger leukocytes occurs in an antigen-independent manner. Under homeostatic conditions, the continuous flow of LPS, the ligand of TLR4, from gut-derived blood is central to the antigen-independent retention of T cells and the deletion of activated CD8^+^ T cells. LSECs not only exert immunoregulatory functions via the selective recruitment of leukocytes but also interact with and activate naïve CD4^+^ and CD8^+^ T cells, mostly favoring the induction of tolerance as opposed to immunity.^46,276–279^

LSECs are further capable of cross-priming CD8^+^ T cells. LSECs can phagocytose, process and present antigens to CD8^+^ T cells on MHC-I,^5^ which drives PD-L1-dependent tolerance in naïve CD8^+^ T cells.^46,280^ However, it is crucial that robust local immunity is generated against harmful pathogens. Accordingly, the cross-priming of increasing concentrations of antigens by LSECs favors effector CD8^+^ T cell differentiation,^277^ possibly due to tonic (or repetitive) TCR stimulation, which may override the inhibitory effect of PD-1 signaling. Additionally, CD8^+^ T cells primed by LSECs rapidly acquire effector GzmB expression prior to T cell entry into the cell cycle. This phenomenon occurs as early as 18 h post CD8^+^ T cell priming and confers robust cytolytic functions. Notably, this acquisition of rapid effector function is not modulated by TCR signaling strength. Instead, IL-6, which is expressed by LSECs, is needed for this unique acquisition of rapid effector functions. Indeed, it was demonstrated that IL-6 signals to primed CD8^+^ T cells, thereby educating primed T cells to respond to proinflammatory cytokines such as type 1 interferon (IFN-I) and hyper IL-6 (i.e., IL-6 complexed to its receptor IL-6R), IFNγ, IL-12 and IL-18, suggesting that cross-priming by LSECs under inflammatory conditions may promote protective immunity against systemic infections.^278^

LSECs are the most profound hepatic cell type known to induce CD25^+^Foxp3^+^ Treg differentiation in naïve CD4^+^ T cells in the presence or absence of exogenous TGF-β. LSECs can also bind latent TGFβ, indicating that these cells can recruit TGFβ from exogenous sources to drive Treg differentiation.^281^ Moreover, following antigen uptake, LSECs produce anti-inflammatory cytokines such as TGFβ and IL-10 to augment the development of antigen-specific tolerance.^282^ In a recent study, LSECs reportedly induced autoreactive recently emigrated CD4^+^ thymic T cells to differentiate into LAG3^+^ Tr1 cells, which lack Foxp3 expression.^279^ The ability of LSECs to induce a unique differentiation program in T cells indicates that these cells can be exploited as preventive and therapeutic targets in allergic and inflammatory diseases. Indeed, LSECs can be targeted by nanoparticles (NPs) in vivo, indicating that NPs can be exploited to specifically deliver antigens to LSECs (Fig. 5c).^283–285^ Indeed, upon myelin oligodendrocyte glycoprotein (MOG) or myelin basic protein (MBP) peptide-induced autoimmune encephalomyelitis (EAE), the delivery of MOG or MBP-loaded NPs to LSECs ameliorated autoimmune encephalomyelitis. A similar outcome was observed when MOG- or MBP-loaded NPs were used as a preventive treatment. In fact, the delivery of autoantigen-loaded NPs after the induction of EAE completely guarded against the development of any clinical disease symptoms.^284^ Interestingly, the induction of tolerance to EAE by NPs also occurred in the absence of the spleen; however, in splenectomized mice, the induced tolerance gradually disappeared, indicating that the spleen is required for the maintenance of liver-induced tolerance. Notably, the protection induced by autoantigen-loaded NPs was dependent on TGFβ signaling in T cells, which is necessary for peripheral Treg generation.^284^ In addition, LSEC-derived Tregs alleviate autoimmune hepatitis.^14^ Similarly, Liu et al.^282^ showed that targeted delivery of biodegradable polymeric polylactic-coglycolic acid allergen-loaded NPs (OVA-NPs) to LSECs in the liver, suppressed ovalbumin (OVA)-induced lung (airway) allergic inflammation. In this study, the authors demonstrated that OVA-NPs delivered with or without mannan or ApoB peptide (ApoBP)-coated ligands for mannose and stabilin receptors, respectively, colocalized preferentially to LSECs. The application of OVA-NPs as a preventive treatment in mice significantly dampened IgE and IgG1 responses to OVA upon allergy induction, accompanied by reductions in the presence of Th2 cytokines IL-4, IL-5 and IL-13, as well as macrophages, and an almost complete absence of eosinophils and neutrophils in bronchoalveolar lavage fluid. Accordingly, the level of TGFβ was increased in OVA-NPs and was highest in ApoB peptide-coated OVA-NPs. Strikingly, ApoBP-coated OVA-NP treatment reduced lung inflammation and mediated Foxp3^+^ Treg accumulation in the lung.^282^ This finding demonstrates that allergen-NPs targeting LSECs could be used as a treatment option for airway allergies. Collectively, these studies described a novel way of exploiting the inherent nature of LSECs as a promising prophylactic and therapeutic approach to allergic and autoimmune diseases.

To overcome the inherent tolerogenicity of LSECs, targeted activation of these cells, which may block tolerance induction, may be necessary to elicit anti-infection and antitumor immunity in the liver. Accordingly, a recent study showed that targeting LSECs in vivo with α-melittin-loaded lipid NPs switched the tolerogenic LSEC phenotype into an activation phenotype.^279^ α-Melittin is a nonselective cytolytic peptide that is water soluble, cationic and amphipathic. It has been suggested that α-melittin exhibits antiviral, antibacterial and antitumor effects.^286^ The α-melittin-loaded NPs specifically localized in LSECs, and there was no adverse effect on liver function, suggesting minimal cytolytic effects.^279^ At the transcriptional level, the most significantly enriched pathways in LSECs after α-melittin-loaded NP treatment were NK cell-mediated cytotoxicity, antigen processing and presentation, chemokine signaling and cytokines involved in leukocyte activation and migration. Subsequent analysis of immune cells in the liver showed increased infiltration of innate and adaptive immune cells such as NK cells, NKT cells, neutrophils, B cells and T cells. The impact of α-melittin-loaded NP administration was evaluated in experimental liver metastasis models using melanoma (B16F10), breast (4T1) and colon (CT26) carcinoma cell lines, and α-melittin NPs consistently induced tumor rejection and enhanced the survival of recipient mice. Detailed analysis of B16F10 tumors from α-melittin NP-injected mice showed enhanced infiltration by mature NK cells and ICOS^+^ Ki67^+^ GzmB^+^ CD8^+^ T cells. α-Melittin NP treatment also promoted the generation of T cell-mediated systemic antitumor memory.^279^ In summary, these reports demonstrate that targeting LSECs with nanoparticles provides a versatile tool to manipulate T cell priming or tolerization in diverse disease contexts.

## Conclusion

Trogocytosis, MHC transfer via extracellular vesicles, and soluble MHCs offer important regulatory mechanisms to enhance antigen presentation in the liver to elicit tolerance or inflammation in AILDs or allograft acceptance or rejection. The transmission of MHC molecules between unrelated APCs in the liver and the cross presentation of exogenous and endogenous antigens enable the education of effector cells across parenchymal barriers, HCs and BECs. The design of disease-relevant pMHC complexes on the surface of nanoparticles or engineered EVs can modulate immune cell activation in a disease-specific manner if disease-specific antigens are known, such as in PBC. Therefore, the identification of disease-related antigens and antibody-secreting B cells supports the development of novel antigen-specific therapeutic tools. Furthermore, the recognition of B cells as immunoregulatory cells that affect T cell effector responses has prompted the identification of distinct phenotypes and distributions of intrahepatic B cells that affect disease activity and progression. Bregs harbor the potential for autologous transfer, as has been developed for Tregs. Additionally, B cell depletion is a potential therapeutic option for refractory or therapy-resistant AILDs and requires further evaluation. GMP-level production of Tregs and CAR-Treg design offer new strategies that will enable the design and generation of novel therapeutic tools for the treatment of AILDs. In addition, the recognition of crosstalk between MAIT cells, iNKT cells and BECs, targeting LSECs via nanoparticles and the relevance of nonpeptide antigens, as well as the identification of regulatory components of the gut-liver axis, will support the development of novel therapeutic strategies.

## References

[1] Carbone M, Neuberger JM (2014). Autoimmune liver disease, autoimmunity and liver transplantation. J. Hepatol..

[2] Than N. N., Oo Y. H. A concise review of autoimmune liver diseases. *Autoimmunity - Pathogenesis, Clinical Aspects and Therapy of Specific Autoimmune Diseases*, (2015).

[3] Carey EJ, Ali AH, Lindor KD (2015). Primary biliary cirrhosis. Lancet.

[4] Karlsen TH, Folseraas T, Thorburn D, Vesterhus M (2017). Primary sclerosing cholangitis - a comprehensive review. J. Hepatol..

[5] Oo YH, Sakaguchi S (2013). Regulatory T-cell directed therapies in liver diseases. J. Hepatol..

[6] Taylor SA, Assis DN, Mack CL (2019). The contribution of B cells in autoimmune liver diseases. Semin. Liver Dis..

[7] Hackstein CP, Klenerman P (2020). Swimming against the current: MAIT cell function is preserved in the peritoneum of advanced liver disease patients. Cell Mol. Gastroenterol. Hepatol..

[8] Horst AK, Neumann K, Diehl L, Tiegs G (2016). Modulation of liver tolerance by conventional and nonconventional antigen-presenting cells and regulatory immune cells. Cell. Mol. Immunol..

[9] Tiegs G, Lohse AW (2010). Immune tolerance: what is unique about the liver. J. Autoimmun..

[10] Calne RY (1969). Induction of immunological tolerance by porcine liver allografts. Nature.

[11] Benseler V (2007). The liver: a special case in transplantation tolerance. Semin. Liver Dis..

[12] Adams DH, Sanchez-Fueyo A, Samuel D (2015). From immunosuppression to tolerance. J. Hepatol..

[13] Grakoui A, Crispe IN (2016). Presentation of hepatocellular antigens. Cell. Mol. Immunol..

[14] Kruse N (2009). Priming of CD4+ T cells by liver sinusoidal endothelial cells induces CD25low forkhead box protein 3- regulatory T cells suppressing autoimmune hepatitis. Hepatology.

[15] Burghardt S (2013). Hepatocytes contribute to immune regulation in the liver by activation of the Notch signaling pathway in T cells. J. Immunol..

[16] Burghardt S, Claass B, Erhardt A, Karimi K, Tiegs G (2014). Hepatocytes induce Foxp3+ regulatory T cells by Notch signaling. J. Leukoc. Biol..

[17] Carambia A (2013). Inhibition of inflammatory CD4 T cell activity by murine liver sinusoidal endothelial cells. J. Hepatol..

[18] Barnes BH (2009). Cholangiocytes as immune modulators in rotavirus-induced murine biliary atresia. Liver Int..

[19] Pinto C, Giordano DM, Maroni L, Marzioni M (2018). Role of inflammation and proinflammatory cytokines in cholangiocyte pathophysiology. Biochim. Biophys. Acta Mol. Basis Dis..

[20] Franco A (1988). Expression of class I and class II major histocompatibility complex antigens on human hepatocytes. Hepatology.

[21] Herkel J (2003). MHC class II-expressing hepatocytes function as antigen-presenting cells and activate specific CD4 T lymphocyutes. Hepatology.

[22] Spengler U (1988). Differential on bile duct expression of MHC class II subregion products epithelial cells and hepatocytes in patients with primary biliary cirrhosis. Hepatology.

[23] Qin B (2013). Association of human leukocyte antigen class II with susceptibility to primary biliary cirrhosis: a systematic review and meta-analysis. PloS ONE.

[24] Zhou G, Ding ZC, Fu J, Levitsky HI (2011). Presentation of acquired peptide-MHC class II ligands by CD4+ regulatory T cells or helper cells differentially regulates antigen-specific CD4+ T cell response. J. Immunol..

[25] Gubser C, Schmaler M, Rossi SW, Palmer E (2016). Monoclonal regulatory T cells provide insights into T cell suppression. Sci. Rep..

[26] Walker LS (2013). Treg and CTLA-4: two intertwining pathways to immune tolerance. J. Autoimmun..

[27] Nakayama M (2014). Antigen presentation by MHC-dressed cells. Front. Immunol..

[28] Kalluri R, LeBleu V (2020). The biology, function, and biomedical applications of exosomes. Science.

[29] Ostman S, Taube M, Telemo E (2005). Tolerosome-induced oral tolerance is MHC dependent. Immunology.

[30] Davies DM (2007). Intercellular transfer of cell-surface proteins is common and can affect many stages of an immune response. Nat. Rev. Immunol..

[31] Oliphant CJ (2014). MHCII-mediated dialog between group 2 innate lymphoid cells and CD4(+) T cells potentiates type 2 immunity and promotes parasitic helminth expulsion. Immunity.

[32] Bal SM, Golebski K, Spits H (2020). Plasticity of innate lymphoid cell subsets. Nat. Rev. Immunol..

[33] Nakayama M (2011). Natural killer (NK)-dendritic cell interactions generate MHC class II-dressed NK cells that regulate CD4+ T cells. Proc. Natl Acad. Sci. USA.

[34] Szabo G, Momen-Heravi F (2017). Extracellular vesicles in liver disease and potential as biomarkers and therapeutic targets. Nat. Rev. Gastroenterol. Hepatol..

[35] Popper H (1962). Mechanism of Cell and Tissue Damage Produced by Immune Reactions.

[36] Wang MX (2001). “Piecemeal” necrosis: renamed troxis necrosis. Exp. Mol. Pathol..

[37] Martinez-Martin N (2011). T cell receptor internalization from the immunological synapse is mediated by TC21 and RhoG GTPase-dependent phagocytosis. Immunity.

[38] French SW (2017). Change in nomenclature for the immunologic synapse from Troxis Necrosis to trogocytosis. Exp. Mol. Pathol..

[39] Miller HW, Suleiman RL, Ralston KS (2019). Trogocytosis by entamoeba histolytica mediates acquisition and display of human cell membrane proteins and evasion of lysis by human serum. mBio.

[40] Scholzel K (2014). Transfer of MHC-class-I molecules among liver sinusoidal cells facilitates hepatic immune surveillance. J. Hepatol..

[41] Carambia A, Herkel J (2014). Liver sinusoidal cells collecting MHC-I molecules: you can’t get enough of a good thing. J. Hepatol..

[42] Knolle PA, Wohlleber D (2016). Immunological functions of liver sinusoidal endothelial cells. Cell. Mol. Immunol..

[43] Bowen DG, McCaughan GW, Bertolino P (2005). Intrahepatic immunity: a tale of two sites?. Trends Immunol..

[44] Tolksdorf F (2018). The PDL1-inducible GTPase Arl4d controls T effector function by limiting IL-2 production. Sci. Rep..

[45] Tay SS (2014). Antigen expression level threshold tunes the fate of CD8 T cells during primary hepatic immune responses. Proc. Natl Acad. Sci. USA.

[46] Diehl L (2008). Tolerogenic maturation of liver sinusoidal endothelial cells promotes B7-homolog 1-dependent CD8+ T cell tolerance. Hepatology.

[47] Pillarisetty VG, Shah AB, Miller G, Bleier JI, DeMatteo RP (2004). Liver dendritic cells are less immunogenic than spleen dendritic cells because of differences in subtype composition. J. Immunol..

[48] Matta BM, Raimondi G, Rosborough BR, Sumpter TL, Thomson AW (2012). IL-27 production and STAT3-dependent upregulation of B7-H1 mediate immune regulatory functions of liver plasmacytoid dendritic cells. J. Immunol..

[49] De Creus A (2005). Low TLR4 expression by liver dendritic cells correlates with reduced capacity to activate allogeneic T cells in response to endotoxin. J. Immunol..

[50] Xia S (2008). Hepatic microenvironment programs hematopoietic progenitor differentiation into regulatory dendritic cells, maintaining liver tolerance. Blood.

[51] Soysa R, Wu X, Crispe IN (2017). Dendritic cells in hepatitis and liver transplantation. Liver Transplant..

[52] Francisco LM (2009). PD-L1 regulates the development, maintenance, and function of induced regulatory T cells. J. Exp. Med..

[53] Ono Y (2018). Graft-infiltrating PD-L1(hi) cross-dressed dendritic cells regulate antidonor T cell responses in mouse liver transplant tolerance. Hepatology.

[54] Londono MC, Rimola A, O’Grady J, Sanchez-Fueyo A (2013). Immunosuppression minimization vs. complete drug withdrawal in liver transplantation. J. Hepatol..

[55] Herrera OB (2004). A novel pathway of alloantigen presentation by dendritic cells. J. Immunol..

[56] Zhuang Q (2016). Graft-infiltrating host dendritic cells play a key role in organ transplant rejection. Nat. Commun..

[57] Hughes AD (2020). Cross-dressed dendritic cells sustain effector T cell responses in islet and kidney allografts. J. Clin. Invest..

[58] Morita M (2010). PD-1/B7-H1 interaction contribute to the spontaneous acceptance of mouse liver allograft. Am. J. Transpl..

[59] Yoshida O (2014). DAP12 deficiency in liver allografts results in enhanced donor DC migration, augmented effector T cell responses and abrogation of transplant tolerance. Am. J. Transpl..

[60] Tokita D (2008). High PD-L1/CD86 ratio on plasmacytoid dendritic cells correlates with elevated T-regulatory cells in liver transplant tolerance. Transplantation.

[61] Davies HS, Pollard SG, Calne RY (1989). Soluble HLA antigens in the circulation of liver graft recipients. Transplantation.

[62] Sumimoto R, Kamada N (1990). Specific suppression of allograft rejection by soluble class I antigen and complexes with monoclonal antibody. Transplantation.

[63] Dolton G (2018). Optimized peptide-MHC multimer protocols for detection and isolation of autoimmune T-cells. Front. Immunol..

[64] Clemente-Casares X (2016). Expanding antigen-specific regulatory networks to treat autoimmunity. Nature.

[65] Umeshappa CS (2019). Suppression of a broad spectrum of liver autoimmune pathologies by single peptide-MHC-based nanomedicines. Nat. Commun..

[66] Christen U, Hintermann E (2018). Autoantibodies in autoimmune hepatitis: can epitopes tell us about the etiology of the disease?. Front. Immunol..

[67] Umeshappa CS (2020). Ubiquitous antigen-specific T regulatory type 1 cells variably suppress hepatic and extrahepatic autoimmunity. J. Clin. Invest.

[68] Hardtke-Wolenski M (2013). Genetic predisposition and environmental danger signals initiate chronic autoimmune hepatitis driven by CD4+ T cells. Hepatology.

[69] Irie J (2006). NOD.c3c4 congenic mice develop autoimmune biliary disease that serologically and pathogenetically models human primary biliary cirrhosis. J. Exp. Med..

[70] Vanderlugt CL, Miller SD (2002). Epitope spreading in immune-mediated diseases: implications for immunotherapy. Nat. Rev. Immunol..

[71] Riedhammer C, Weissert R (2015). Antigen presentation, autoantigens, and immune regulation in multiple sclerosis and other autoimmune diseases. Front. Immunol..

[72] Alonso R (2005). Diacylglycerol kinase alpha regulates the secretion of lethal exosomes bearing Fas ligand during activation-induced cell death of T lymphocytes. J. Biol. Chem..

[73] Muntasell A, Berger AC, Roche PA (2007). T cell-induced secretion of MHC class II-peptide complexes on B cell exosomes. EMBO J..

[74] Shelke GV (2019). Endosomal signalling via exosome surface TGFbeta-1. J. Extracell. Vesicles.

[75] Mastoridis S, Sanchez-Fueyo A, Martinez-Llordella M (2018). Following clinical liver transplantation, the majority of circulating cells exhibiting donor MHC are “cross-dressed” not “passenger” leukocytes. J. Hepatol..

[76] Marino J (2016). Donor exosomes rather than passenger leukocytes initiate alloreactive T cell responses after transplantation. Sci. Immunol..

[77] Zeng F, Morelli AE (2018). Extracellular vesicle-mediated MHC cross-dressing in immune homeostasis, transplantation, infectious diseases, and cancer. Semin. Immunopathol..

[78] Benichou G, Wang M, Ahrens K, Madsen JC (2020). Extracellular vesicles in allograft rejection and tolerance. Cell Immunol..

[79] Nazimek K, Bryniarski K (2020). Approaches to inducing antigen-specific immune tolerance in allergy and autoimmunity: Focus on antigen-presenting cells and extracellular vesicles. Scand. J. Immunol..

[80] Wen J, Friedman JR (2012). miR-122 regulates hepatic lipid metabolism and tumor suppression. J. Clin. Invest..

[81] Esau C (2006). miR-122 regulation of lipid metabolism revealed by in vivo antisense targeting. Cell Metab..

[82] Castoldi M (2011). The liver-specific microRNA miR-122 controls systemic iron homeostasis in mice. J. Clin. Investig..

[83] Conde-Vancells J (2010). Candidate biomarkers in exosome-like vesicles purified from rat and mouse urine samples. Proteom. Clin. Appl.

[84] Welker MW (2012). Soluble serum CD81 is elevated in patients with chronic hepatitis C and correlates with alanine aminotransferase serum activity. PloS ONE.

[85] Harris HJ (2010). Claudin association with CD81 defines hepatitis C virus entry. J. Biol. Chem..

[86] Yanez-Mo M, Barreiro O, Gordon-Alonso M, Sala-Valdes M, Sanchez-Madrid F (2009). Tetraspanin-enriched microdomains: a functional unit in cell plasma membranes. Trends Cell Biol..

[87] Sagi Y, Landrigan A, Levy R, Levy S (2012). Complementary costimulation of human T-cell subpopulations by cluster of differentiation 28 (CD28) and CD81. Proc. Natl Acad. Sci. USA.

[88] Li S, Li S, Wu S, Chen L (2019). Exosomes modulate the viral replication and host immune responses in HBV infection. Biomed. Res. Int..

[89] Cobb DA, Kim OK, Golden-Mason L, Rosen HR, Hahn YS (2018). Hepatocyte-derived exosomes promote T follicular regulatory cell expansion during hepatitis C virus infection. Hepatology.

[90] Li J (2013). Exosomes mediate the cell-to-cell transmission of IFN-alpha-induced antiviral activity. Nat. Immunol..

[91] Kornek M (2012). Circulating microparticles as disease-specific biomarkers of severity of inflammation in patients with hepatitis C or nonalcoholic steatohepatitis. Gastroenterology.

[92] Ibrahim SH (2016). Mixed lineage kinase 3 mediates release of C-X-C motif ligand 10-bearing chemotactic extracellular vesicles from lipotoxic hepatocytes. Hepatology.

[93] Mann DA, Smart DE (2002). Transcriptional regulation of hepatic stellate cell activation. Gut.

[94] Chen L, Chen R, Kemper S, Charrier A, Brigstock DR (2015). Suppression of fibrogenic signaling in hepatic stellate cells by Twist1-dependent microRNA-214 expression: Role of exosomes in horizontal transfer of Twist1. Am. J. Physiol. Gastrointest. Liver Physiol..

[95] Wiklander OP (2015). Extracellular vesicle in vivo biodistribution is determined by cell source, route of administration and targeting. J. Extracell. Vesicles.

[96] Bala S (2015). Biodistribution and function of extracellular miRNA-155 in mice. Sci. Rep..

[97] Bala S (2016). The pro-inflammatory effects of miR-155 promote liver fibrosis and alcohol-induced steatohepatitis. J. Hepatol..

[98] Csak T (2015). MicroRNA-155 deficiency attenuates liver steatosis and fibrosis without reducing inflammation in a mouse model of steatohepatitis. PloS ONE.

[99] Jordan P, Kübler D (1996). Autoimmune diseases: nuclear autoantigens can be found at the cell-surface. Mol. Biol. Rep..

[100] Liver EAftSot. (2017). EASL Clinical Practice Guidelines: the diagnosis and management of patients with primary biliary cholangitis. J. Hepatol..

[101] Sebode M, Weiler-Normann C, Liwinski T, Schramm C (2018). Autoantibodies in autoimmune liver disease-clinical and diagnostic relevance. Front. Immunol..

[102] Liberal R, Grant C, Sakkas L, Bizzaro N, Bogdanos DP (2013). Diagnostic and clinical significance of anti-centromere antibodies in primary biliary cirrhosis. Clin. Res. Hepatol. Gastroenterol..

[103] Honda A, Ikegami T, Matsuzaki Y (2015). Anti‐gp210 and anti‐centromere antibodies for the prediction of PBC patients with an incomplete biochemical response to UDCA and bezafibrate. Hepataol. Res..

[104] Couto CA (2014). Antismooth muscle and antiactin antibodies are indirect markers of histological and biochemical activity of autoimmune hepatitis. Hepatology.

[105] Kanzler S (1999). Clinical significance of autoantibodies to soluble liver antigen in autoimmune hepatitis. J. Hepatol..

[106] Ballot E, Homberg JC, Johanet C (2000). Antibodies to soluble liver antigen: an additional marker in type 1 auto-immune hepatitis. J. Hepatol..

[107] Czaja A, Donaldson PT, Lohse AW (2002). Antibodies to soluble liver antigen/liver pancreas and Hla risk factors for type 1 autoimmune hepatitis. Am. J. Gastroenterol..

[108] Mix H (2008). Identification of CD4 T-cell epitopes in soluble liver antigen/liver pancreas autoantigen in autoimmune hepatitis. Gastroenterology.

[109] Ma Y (2006). Polyclonal T-cell responses to cytochrome P450IID6 are associated with disease activity in autoimmune hepatitis type 2. Gastroenterology.

[110] Longhi MS (2007). Cytochrome P450IID6-specific CD8 T cell immune responses mirror disease activity in autoimmune hepatitis type 2. Hepatology.

[111] Löhr H (1990). The human hepatic asialoglycoprotein receptor is a target antigen for liver-infiltrating T cells in autoimmune chronic active hepatitis and primary biliary cirrhosis. Hepatology.

[112] Braun S, Berg C, Buck S, Gregor M, Klein R (2010). Catalytic domain of PDC-E2 contains epitopes recognized by antimitochondrial antibodies in primary biliary cirrhosis. World J. Gastroenterol..

[113] Rigopoulou EI (2007). Antimitochondrial antibodies of immunoglobulin G3 subclass are associated with a more severe disease course in primary biliary cirrhosis. Liver Int..

[114] Mantis NJ, Rol N, Corthesy B (2011). Secretory IgA’s complex roles in immunity and mucosal homeostasis in the gut. Mucosal Immunol..

[115] Inamine T, Schnabl B (2017). Immunoglobulin A and liver diseases. J. Gastroenterol..

[116] Oldstone MB (2014). Molecular mimicry: its evolution from concept to mechanism as a cause of autoimmune diseases. Monoclon. Antib. Immunodiagn. Immunother..

[117] Christen U, von Herrath MG (2004). Induction, acceleration or prevention of autoimmunity by molecular mimicry. Mol. Immunol..

[118] Lehmann PV, Forsthuber T, Miller A, Sercarz EE (1992). Spreading of T-cell autoimmunity to cryptic determinants of an autoantigen. Nature.

[119] Christen U (2019). Pathogen infection and autoimmune disease. Clin. Exp. Immunol..

[120] Xu B, Broome U, Ericzon B-G, Sumitran-Holgersson S (2002). High frequency of autoantibodies in patients with primary sclerosing cholangitis that bind biliary epithelial cells and induce expression of CD44 and production of interleukin 6. Gut.

[121] Mandal A (1994). Autoantibodies in sclerosing cholangitis against a shared peptide in biliary and colon epithelium. Gastroenterology.

[122] Berglin L, Bjorkstrom NK, Bergquist A (2013). Primary sclerosing cholangitis is associated with autoreactive IgA antibodies against biliary epithelial cells. Scand. J. Gastroenterol..

[123] Karrar A (2007). Biliary epithelial cell antibodies link adaptive and innate immune responses in primary sclerosing cholangitis. Gastroenterology.

[124] Jeffery HC (2019). Bidirectional cross-talk between biliary epithelium and Th17 cells promotes local Th17 expansion and bile duct proliferation in biliary liver diseases. J. Immunol..

[125] Tornai T (2018). Loss of tolerance to gut immunity protein, glycoprotein 2 (GP2) is associated with progressive disease course in primary sclerosing cholangitis. Sci. Rep..

[126] Papp M (2015). Rediscovery of the anti-pancreatic antibodies and evaluation of their prognostic value in a prospective clinical cohort of Crohn’s patients: the importance of specific target antigens [GP2 and CUZD1]. J. Crohns Colitis.

[127] Chung BK (2017). Phenotyping and auto-antibody production by liver-infiltrating B cells in primary sclerosing cholangitis and primary biliary cholangitis. J. Autoimmun..

[128] Foureau DM (2015). Comparative analysis of portal hepatic infiltrating leucocytes in acute drug-induced liver injury, idiopathic autoimmune and viral hepatitis. Clin. Exp. Immunol..

[129] Abe K (2016). Interleukin-21 plays a critical role in the pathogenesis and severity of type I autoimmune hepatitis. Springerplus.

[130] Ma L, Qin J, Ji H, Zhao P, Jiang Y (2014). Tfh and plasma cells are correlated with hypergammaglobulinaemia in patients with autoimmune hepatitis. Liver Int..

[131] Aoki N (2011). Dysregulated generation of follicular helper T cells in the spleen triggers fatal autoimmune hepatitis in mice. Gastroenterology.

[132] Oo YH (2012). CXCR3-dependent recruitment and CCR6-mediated positioning of Th-17 cells in the inflamed liver. J. Hepatol..

[133] Katz SL, Parker D, Turk JL (1974). B-cell suppression of delayed hypersensitivity reactions. Nature.

[134] Yanaba K (2008). A regulatory B cell subset with a unique CD1dhiCD5+ phenotype controls T cell-dependent inflammatory responses. Immunity.

[135] Wortel CM, Heidt S (2017). Regulatory B cells: Phenotype, function and role in transplantation. Transpl. Immunol..

[136] Chong AS, Khiew SH (2017). Transplantation tolerance: don’t forget about the B cells. Clin. Exp. Immunol..

[137] Norris S (1998). Resident human hepatic lymphocytes are phenotypically different from circulating lymphocytes. J. Hepatol..

[138] Xiao S, Brooks CR, Sobel RA, Kuchroo VK (2015). Tim-1 is essential for induction and maintenance of IL-10 in regulatory B cells and their regulation of tissue inflammation. J. Immunol..

[139] Yanaba K (2011). IL-10-producing regulatory B10 cells inhibit intestinal injury in a mouse model. Am. J. Pathol..

[140] Oka A (2014). Role of regulatory B cells in chronic intestinal inflammation: association with pathogenesis of Crohn’s disease. Inflamm. Bowel Dis..

[141] Yang M (2012). IL-10-producing regulatory B10 cells ameliorate collagen-induced arthritis via suppressing Th17 cell generation. Am. J. Pathol..

[142] Hamad ARA, Ahmed R, Thomas Donner T, Fousteri G (2016). B cell targeted immunotherapy for type 1 diabetes: What can make it work?. Discov. Med.

[143] Watanabe R (2010). Regulatory B cells (B10 cells) have a suppressive role in murine lupus: CD19 and B10 cell deficiency exacerbates systemic autoimmunity. J. Immunol..

[144] Yang M, Rui K, Wang S, Lu L (2013). Regulatory B cells in autoimmune diseases. Cell. Mol. Immunol..

[145] Tabarkiewicz J, Pogoda K, Karczmarczyk A, Pozarowski P, Giannopoulos K (2015). The role of IL-17 and Th17 lymphocytes in autoimmune diseases. Arch. Immunol. Ther. Exp..

[146] Abadja F, Sarraj B, Ansari MJ (2012). Significance of T helper 17 immunity in transplantation. Curr. Opin. Organ Transpl..

[147] Dominguez-Villar M, Hafler DA (2018). Regulatory T cells in autoimmune disease. Nat. Immunol..

[148] Tian J (2001). Lipopolysaccharide-activated B cells down-regulate Th1 immunity and prevent autoimmune diabetes in nonobese diabetic mice. J. Immunol..

[149] van de Veen W (2013). IgG4 production is confined to human IL-10-producing regulatory B cells that suppress antigen-specific immune responses. J. allergy Clin. Immunol..

[150] Hasan MM (2019). CD24(hi)CD38(hi) and CD24(hi)CD27(+) human regulatory B cells display common and distinct functional characteristics. J. Immunol..

[151] Dwyer KM (2010). Expression of CD39 by human peripheral blood CD4+ CD25+ T cells denotes a regulatory memory phenotype. Am. J. Transpl..

[152] Li J (2017). CD39/CD73 upregulation on myeloid-derived suppressor cells via TGF-beta-mTOR-HIF-1 signaling in patients with non-small cell lung cancer. Oncoimmunology.

[153] Chen Q (2020). CD19(+)CD24(hi)CD38(hi) B cell dysfunction in primary biliary cholangitis. Mediators Inflamm..

[154] Blair PA (2010). CD19(+)CD24(hi)CD38(hi) B cells exhibit regulatory capacity in healthy individuals but are functionally impaired in systemic Lupus Erythematosus patients. Immunity.

[155] Xiao S (2012). Defect in regulatory B-cell function and development of systemic autoimmunity in T-cell Ig mucin 1 (Tim-1) mucin domain-mutant mice. Proc. Natl Acad. Sci. USA.

[156] Ding Q (2011). Regulatory B cells are identified by expression of TIM-1 and can be induced through TIM-1 ligation to promote tolerance in mice. J. Clin. Invest..

[157] Yeung MY (2015). TIM-1 signaling is required for maintenance and induction of regulatory B cells. Am. J. Transpl..

[158] Degauque N (2008). Immunostimulatory Tim-1-specific antibody deprograms Tregs and prevents transplant tolerance in mice. J. Clin. Invest..

[159] Umetrsu SE (2005). TIM-1 induces T cell activation and inhibits the development of peripheral tolerance. Nat. Immunol..

[160] Xiao S (2007). Differential engagement of Tim-1 during activation can positively or negatively costimulate T cell expansion and effector function. J. Exp. Med..

[161] Ueno T (2008). The emerging role of T cell Ig mucin 1 in alloimmune responses in an experimental mouse transplant model. J. Clin. Invest..

[162] Liu X (2015). B cells expressing CD11b effectively inhibit CD4+ T-cell responses and ameliorate experimental autoimmune hepatitis in mice. Hepatology.

[163] Lin X (2019). IL-10-producing regulatory B cells restrain the T follicular helper cell response in primary Sjogren’s syndrome. Cell. Mol. Immunol..

[164] Lee KM (2014). TGF-beta-producing regulatory B cells induce regulatory T cells and promote transplantation tolerance. Eur. J. Immunol..

[165] Mauri C, Menon M (2017). Human regulatory B cells in health and disease: therapeutic potential. J. Clin. Invest..

[166] Yanaba K (2007). B cell depletion delays collagen-induced arthritis in mice: arthritis induction requires synergy between humoral and cell-mediated immunity. J. Immunol..

[167] Gray M, Miles K, Salter D, Gray D, Savill J (2007). Apoptotic cells protect mice from autoimmune inflammation by the induction of regulatory B cells. Proc. Natl Acad. Sci. USA.

[168] Berghen N, Vusteke J-B, Westhovens R, Lenaerts J, de Langhe E (2018). Rituximab in systemic autoimmune rheumatic diseases: indications and practical use. Acta Clin. Belg..

[169] Kaegi C (2019). Systematic review of safety and efficacy of rituximab in treating immune-mediated disorders. Front. Immunol..

[170] Pierpont TM, Limper CB, Richards KL (2018). Past, present, and future of rituximab-the world’s first oncology monoclonal antibody therapy. Front. Oncol..

[171] D’Agostino D, Costaguta A, Alvarez F (2013). Successful treatment of refractory autoimmune hepatitis with rituximab. Pediatrics.

[172] Burak KW (2013). Rituximab for the treatment of patients with autoimmune hepatitis who are refractory or intolerant to standard therapy. Gastroenterology.

[173] Than NN (2019). Efficacy of rituximab in difficult-to-manage autoimmune hepatitis: results from the International Autoimmune Hepatitis Group. JHEP Rep..

[174] Tan YG (2016). Clonal characteristics of circulating B lymphocyte repertoire in primary biliary cholangitis. J. Immunol..

[175] Tsuda M (2012). Biochemical and immunologic effects of rituximab in patients with primary biliary cirrhosis and an incomplete response to ursodeoxycholic acid. Hepatology.

[176] Schneider P (1999). BAFF, a novel ligand of the tumor necrosis factor family, stimulates B cell growth. J. Exp. Med..

[177] Thompson JS (2001). BAFF-R, a Newly Identified TNF Receptor That Specifically Interacts with BAFF. Science.

[178] Boule MW (2004). Toll-like receptor 9-dependent and -independent dendritic cell activation by chromatin-immunoglobulin G complexes. J. Exp. Med..

[179] Mackay F, Schneider P (2009). Cracking the BAFF code. Nat. Rev. Immunol..

[180] Groom JR (2007). BAFF and MyD88 signals promote a lupuslike disease independent of T cells. J. Exp. Med..

[181] Mackay F, Sierro F, Grey ST, Gordon TP (2005). The BAFF/APRIL system: an important player in systemic rheumatic diseases. Curr. Dir. Autoimmun..

[182] Thien M (2004). Excess BAFF rescues self-reactive B cells from peripheral deletion and allows them to enter forbidden follicular and marginal zone niches. Immunity.

[183] Ma N (2017). B cell activating factor (BAFF) selects IL-10(-)B cells over IL-10(+)B cells during inflammatory responses. Mol. Immunol..

[184] Arvaniti P (2020). Belimumab is a promising third-line treatment option for refractory autoimmune hepatitis. JHEP Rep..

[185] Atif M, Warner S, Oo YH (2018). Linking the gut and liver: crosstalk between regulatory T cells and mucosa-associated invariant T cells. Hepatol. Int..

[186] Kjer-Nielsen L (2012). MR1 presents microbial vitamin B metabolites to MAIT cells. Nature.

[187] Treiner E (2003). Selection of evolutionarily conserved mucosal-associated invariant T cells by MR1. Nature.

[188] Reantragoon R (2013). Antigen-loaded MR1 tetramers define T cell receptor heterogeneity in mucosal-associated invariant T cells. J. Exp. Med..

[189] Le Bourhis L (2010). Antimicrobial activity of mucosal-associated invariant T cells. Nat. Immunol..

[190] Toubal A, Nel I, Lotersztajn S, Lehuen A (2019). Mucosal-associated invariant T cells and disease. Nat. Rev. Immunol..

[191] Lamichhane R (2019). TCR- or cytokine-activated CD8(+) mucosal-associated invariant T cells are rapid polyfunctional effectors that can coordinate immune responses. Cell Rep..

[192] van Wilgenburg B (2018). MAIT cells contribute to protection against lethal influenza infection in vivo. Nat. Commun..

[193] Loh L (2016). Human mucosal-associated invariant T cells contribute to antiviral influenza immunity via IL-18-dependent activation. Proc. Natl Acad. Sci. USA.

[194] Ussher JE, Willberg CB, Klenerman P (2018). MAIT cells and viruses. Immunol. Cell Biol..

[195] Hinks TS (2016). Steroid-induced deficiency of mucosal-associated invariant T cells in the chronic obstructive pulmonary disease lung. Implications for nontypeable haemophilus influenzae infection. Am. J. Respiratory Crit. Care Med..

[196] Hinks T (2015). Multidimensional endotypes of asthma: topological data analysis of cross-sectional clinical, pathological, and immunological data. Lancet.

[197] Jeffery HC (2016). Biliary epithelium and liver B cells exposed to bacteria activate intrahepatic MAIT cells through MR1. J. Hepatol..

[198] Heydtmann M (2005). CXC chemokine ligand 16 promotes integrin-mediated adhesion of liver-infiltrating lymphocytes to cholangiocytes and hepatocytes within the inflamed human liver. J. Immunol..

[199] von Seth E (2018). Primary sclerosing cholangitis leads to dysfunction and loss of MAIT cells. Eur. J. Immunol..

[200] Riva A (2018). Mucosa-associated invariant T cells link intestinal immunity with antibacterial immune defects in alcoholic liver disease. Gut.

[201] Berkhout L (2019). Deletion of tumour necrosis factor alpha receptor 1 elicits an increased TH17 immune response in the chronically inflamed liver. Sci. Rep..

[202] Tan Z (2013). IL-17A plays a critical role in the pathogenesis of liver fibrosis through hepatic stellate cell activation. J. Immunol..

[203] Bottcher K (2018). MAIT cells are chronically activated in patients with autoimmune liver disease and promote profibrogenic hepatic stellate cell activation. Hepatology.

[204] Hegde P (2018). Mucosal-associated invariant T cells are a profibrogenic immune cell population in the liver. Nat. Commun..

[205] Berkson JD, Prlic M (2017). The MAIT conundrum - how human MAIT cells distinguish bacterial colonization from infection in mucosal barrier tissues. Immunol. Lett..

[206] Wakao H, Sugimoto C, Kimura S, Wakao R (2017). Mucosal-associated invariant T cells in regenerative medicine. Front. Immunol..

[207] Wakao H (2013). Expansion of functional human mucosal-associated invariant T cells via reprogramming to pluripotency and redifferentiation. Cell Stem Cell.

[208] Darmoise A (2010). Lysosomal alpha-galactosidase controls the generation of self lipid antigens for natural killer T cells. Immunity.

[209] Brennan PJ, Brigl M, Brenner MB (2013). Invariant natural killer T cells: an innate activation scheme linked to diverse effector functions. Nat. Rev. Immunol..

[210] Kita H (2002). Quantitation and phenotypic analysis of natural killer T cells in primary biliary cirrhosis using a human CD1d tetramer. Gastroenterology.

[211] Coquet JM (2008). Diverse cytokine production by NKT cell subsets and identification of an IL-17-producing CD4-NK1.1- NKT cell population. Proc. Natl Acad. Sci. USA.

[212] Schrumpf E (2015). The biliary epithelium presents antigens to and activates natural killer T cells. Hepatology.

[213] Tsuneyama K (1998). Increased CD1d expression on small bile duct epithelium and epithelioid granuloma in livers in primary biliary cirrhosis. Hepatology.

[214] Kaneko Y (2000). Augmentation of Va14 NKT cell–mediated cytotoxicity by interleukin 4 in an autocrine mechanism resulting in the development of concanavalin A–induced hepatitis. J. Exp. Med..

[215] Wondimu Z, Santodomingo-Garzon T, Le T, Swain MG (2010). Protective role of interleukin-17 in murine NKT cell-driven acute experimental hepatitis. Am. J. Pathol..

[216] Biburger M, Tiegs G (2005). Galactosylceramide-induced liver injury in mice is mediated by TNF- but independent of Kupffer cells. J. Immunol..

[217] Berntsen NL (2018). Natural killer T cells mediate inflammation in the bile ducts. Mucosal Immunol..

[218] Mattner J (2008). Liver autoimmunity triggered by microbial activation of natural killer T cells. Cell Host Microbe.

[219] Derkow K (2007). Differential priming of CD8 and CD4 T-cells in animal models of autoimmune hepatitis and cholangitis. Hepatology.

[220] Glaser F (2019). Liver infiltrating T cells regulate bile acid metabolism in experimental cholangitis. J. Hepatol..

[221] Fiorucci S, Biagioli M, Zampella A, Distrutti E (2018). Bile acids activated receptors regulate innate immunity. Front. Immunol..

[222] Sebode M (2014). Reduced FOXP3(+) regulatory T cells in patients with primary sclerosing cholangitis are associated with IL2RA gene polymorphisms. J. Hepatol..

[223] Peiseler M (2012). FOXP3+ regulatory T cells in autoimmune hepatitis are fully functional and not reduced in frequency. J. Hepatol..

[224] Ferri S (2010). A multifaceted imbalance of T cells with regulatory function characterizes type 1 autoimmune hepatitis. Hepatology.

[225] Buckner JH (2010). Mechanisms of impaired regulation by CD4(+)CD25(+)FOXP3(+) regulatory T cells in human autoimmune diseases. Nat. Rev. Immunol..

[226] Karkhah A, Javanian M, Ebrahimpour S (2018). The role of regulatory T cells in immunopathogenesis and immunotherapy of viral infections. Infect. Genet. Evol..

[227] Boettler T (2005). T cells with a CD4+CD25+ regulatory phenotype suppress in vitro proliferation of virus-specific CD8+ T cells during chronic hepatitis C virus infection. J. Virol..

[228] Franzese O (2005). Modulation of the CD8+-T-cell response by CD4+ CD25+ regulatory T cells in patients with hepatitis B virus infection. J. Virol..

[229] Lu C (2019). Current perspectives on the immunosuppressive tumor microenvironment in hepatocellular carcinoma: challenges and opportunities. Mol. Cancer.

[230] Seddiki N (2006). Expression of interleukin (IL)-2 and IL-7 receptors discriminates between human regulatory and activated T cells. J. Exp. Med..

[231] Chen YY (2016). Human intrahepatic regulatory T cells are functional, require IL-2 from effector cells for survival, and are susceptible to Fas ligand-mediated apoptosis. Hepatology.

[232] Wing K (2008). CTLA-4 control over Foxp3+ regulatory T cell function. Science.

[233] Deaglio S (2007). Adenosine generation catalyzed by CD39 and CD73 expressed on regulatory T cells mediates immune suppression. J. Exp. Med..

[234] Chinen T (2016). An essential role for the IL-2 receptor in Treg cell function. Nat. Immunol..

[235] Ghiringhelli F (2005). CD4+CD25+ regulatory T cells inhibit natural killer cell functions in a transforming growth factor-beta-dependent manner. J. Exp. Med..

[236] Schmidt A, Oberle N, Krammer PH (2012). Molecular mechanisms of treg-mediated T cell suppression. Front. Immunol..

[237] Owen DL, Farrar MA (2017). STAT5 and CD4 + T Cell Immunity. F1000Res.

[238] Levine AG, Arvey A, Jin W, Rudensky AY (2014). Continuous requirement for the TCR in regulatory T cell function. Nat. Immunol..

[239] Chen W (2003). Conversion of peripheral CD4+CD25- naive T cells to CD4+CD25+ regulatory T cells by TGF-beta induction of transcription factor Foxp3. J. Exp. Med..

[240] Shevach EM (2012). Application of IL-2 therapy to target T regulatory cell function. Trends Immunol..

[241] Wan YY, Flavell RA (2007). Regulatory T-cell functions are subverted and converted owing to attenuated Foxp3 expression. Nature.

[242] Xu L, Kitani A, Fuss I, Strober W (2007). Cutting edge: regulatory T cells induce CD4+CD25-Foxp3- T cells or are self-induced to become Th17 cells in the absence of exogenous TGF-beta. J. Immunol..

[243] Longhi MS (2011). Autoantigen-specific regulatory T cells, a potential tool for immune-tolerance reconstitution in type-2 autoimmune hepatitis. Hepatology.

[244] Hu CJ (2012). Identification of new autoantigens for primary biliary cirrhosis using human proteome microarrays. Mol. Cell. Proteomics.

[245] Jeffery HC, Kaur Braitch M, Brown S, Oo YH (2016). Clinical potential of regulatory T cell therapy in liver diseases: an overview and current perspectives. Front. Immunol..

[246] Oo YH (2019). Liver homing of clinical grade Tregs after therapeutic infusion in patients with autoimmune hepatitis. JHEP Rep..

[247] Romano M, Fanelli G, Albany CJ, Giganti G, Lombardi G (2019). Past, present, and future of regulatory T cell therapy in transplantation and autoimmunity. Front. Immunol..

[248] Pol JG, Caudana P, Paillet J, Piaggio E, Kroemer G (2020). Effects of interleukin-2 in immunostimulation and immunosuppression. J. Exp. Med..

[249] Letourneau S (2010). IL-2/anti-IL-2 antibody complexes show strong biological activity by avoiding interaction with IL-2 receptor alpha subunit CD25. Proc. Natl Acad. Sci. USA.

[250] Saadoun D (2011). Regulatory T-cell responses to low-dose interleukin-2 in HCV-induced vasculitis. N. Engl. J. Med..

[251] Jeffery LE (2009). 1,25-Dihydroxyvitamin D3 and IL-2 combine to inhibit T cell production of inflammatory cytokines and promote development of regulatory T cells expressing CTLA-4 and FoxP3. J. Immunol..

[252] Scotta C (2013). Differential effects of rapamycin and retinoic acid on expansion, stability and suppressive qualities of human CD4(+)CD25(+)FOXP3(+) T regulatory cell subpopulations. Haematologica.

[253] Golovina TN (2011). Retinoic acid and rapamycin differentially affect and synergistically promote the ex vivo expansion of natural human T regulatory cells. PloS ONE.

[254] Fraser H (2018). A rapamycin-based GMP-compatible process for the isolation and expansion of regulatory T cells for clinical trials. Mol. Ther. Methods Clin. Dev..

[255] Erhardt A, Biburger M, Papadopoulos T, Tiegs G (2007). IL-10, regulatory T cells, and Kupffer cells mediate tolerance in concanavalin A-induced liver injury in mice. Hepatology.

[256] Lapierre P, Beland K, Yang R, Alvarez F (2013). Adoptive transfer of ex vivo expanded regulatory T cells in an autoimmune hepatitis murine model restores peripheral tolerance. Hepatology.

[257] Erhardt A (2011). CXCR3 deficiency exacerbates liver disease and abrogates tolerance in a mouse model of immune-mediated hepatitis. J. Immunol..

[258] Hasegawa H (2008). Therapeutic effect of CXCR3-expressing regulatory T cells on liver, lung and intestinal damages in a murine acute GVHD model. Gene Ther..

[259] MacDonald KG (2016). Alloantigen-specific regulatory T cells generated with a chimeric antigen receptor. J. Clin. Invest..

[260] Fritsche E, Volk HD, Reinke P, Abou-El-Enein M (2020). Toward an optimized process for clinical manufacturing of CAR-Treg cell therapy. Trends Biotechnol..

[261] Nowak A (2018). CD137+CD154- expression as a regulatory T cell (Treg)-specific activation signature for identification and sorting of stable human Tregs from in vitro expansion cultures. Front. Immunol..

[262] Dawson NAJ (2020). Functional effects of chimeric antigen receptor co-receptor signaling domains in human regulatory T cells. Sci. Transl. Med..

[263] Brunstein CG (2011). Infusion of ex vivo expanded T regulatory cells in adults transplanted with umbilical cord blood: safety profile and detection kinetics. Blood.

[264] Marek-Trzonkowska N (2012). Administration of CD4+CD25highCD127- regulatory T cells preserves beta-cell function in type 1 diabetes in children. Diabetes Care.

[265] Bluestone JA (2015). Type 1 diabetes immunotherapy using polyclonal regulatory T cells. Sci. Transl. Med..

[266] Trzonkowski P (2009). First-in-man clinical results of the treatment of patients with graft versus host disease with human ex vivo expanded CD4+CD25+CD127− T regulatory cells. Clin. Immunol..

[267] Todo S (2016). A pilot study of operational tolerance with a regulatory T-cell-based cell therapy in living donor liver transplantation. Hepatology.

[268] Borsellino G (2007). Expression of ectonucleotidase CD39 by Foxp3+ Treg cells: hydrolysis of extracellular ATP and immune suppression. Blood.

[269] Tai X (2012). Basis of CTLA-4 function in regulatory and conventional CD4(+) T cells. Blood.

[270] Lafoz E, Ruart M, Anton A, Oncins A, Hernandez-Gea V (2020). The endothelium as a driver of liver fibrosis and regeneration. Cells.

[271] Jenne CN, Kubes P (2013). Immune surveillance by the liver. Nat. Immunol..

[272] Chen GY, Nunez G (2010). Sterile inflammation: sensing and reacting to damage. Nat. Rev. Immunol..

[273] Shetty S, Lalor PF, Adams DH (2018). Liver sinusoidal endothelial cells - gatekeepers of hepatic immunity. Nat. Rev. Gastroenterol. Hepatol..

[274] Canton J, Neculai D, Grinstein S (2013). Scavenger receptors in homeostasis and immunity. Nat. Rev. Immunol..

[275] Patten DA (2017). Human liver sinusoidal endothelial cells promote intracellular crawling of lymphocytes during recruitment: a new step in migration. Hepatology.

[276] Böttcher JP (2013). Liver-primed memory T cells generated under noninflammatory conditions provide anti-infectious immunity. Cell Rep..

[277] Schurich A (2010). Dynamic regulation of CD8 T cell tolerance induction by liver sinusoidal endothelial cells. J. Immunol..

[278] Böttcher JP (2014). IL-6 trans-signaling-dependent rapid development of cytotoxic CD8+ T cell function. Cell Rep..

[279] Xu X (2016). Liver sinusoidal endothelial cells induce tolerance of autoreactive CD4+ recent thymic emigrants. Sci. Rep..

[280] Limmer A (2005). Cross-presentation of oral antigens by liver sinusoidal endothelial cells leads to CD8 T cell tolerance. Eur. J. Immunol..

[281] Carambia A (2014). TGF-β-dependent induction of CD4+CD25+Foxp3 + Tregs by liver sinusoidal endothelial cells. J. Hepatol..

[282] Liu Q (2019). Use of polymeric nanoparticle platform targeting the liver to induce Treg-mediated antigen-specific immune tolerance in a pulmonary allergen sensitization model. ACS Nano.

[283] Marquez J (2018). Targeting liver sinusoidal endothelial cells with miR-20a-loaded nanoparticles reduces murine colon cancer metastasis to the liver. Int. J. Cancer.

[284] Carambia A (2015). Nanoparticle-based autoantigen delivery to Treg-inducing liver sinusoidal endothelial cells enables control of autoimmunity in mice. J. Hepatol..

[285] Yu X (2019). Immune modulation of liver sinusoidal endothelial cells by melittin nanoparticles suppresses liver metastasis. Nat. Commun..

[286] Rady I, Siddiqui IA, Rady M, Mukhtar H (2017). Melittin, a major peptide component of bee venom, and its conjugates in cancer therapy. Cancer Lett..

